# Supplementary material for: What outcomes are important to families with a lived experience of stillbirth? A qualitative study to inform the development of a core outcome set for stillbirth care
Source: PLoS One. 2026 May 19;21(5):e0347544. doi: 10.1371/journal.pone.0347544 (PMC13186333; doi:10.1371/journal.pone.0347544)
Supplement: S1 Table — (DOCX) [file pone.0347544.s001.docx]

**Supporting Information Table 1: Information power dimensions for stillbirth care research interviews^12^**

| **Information power dimension** | **Comments** |
| --- | --- |
| **Study aim** | Broad aim thus large purposive sample to gain diverse sample of parents. |
| **Sample specificity** | Highly specific population in alignment with the study aims. |
| **Use of established theory** | Study influenced by grounded theory, however the research team had knowledge about stillbirth care outcomes from a prior literature review^3^. |
| **Quality of dialogue** | Strong communication between participant and researcher, lived experiences shared and stillbirth care discussed. At times interview conversation was unfocused on outcomes of stillbirth care specifically. Researcher needed to ‘tease’ out outcomes by using examples of care described by parents. More interviews needed to be conducted to consolidate outcomes. Data obtained were dense with a large number of quotes to exemplify outcomes. |
| **Analysis strategy** | An exploratory cross-case analysis was undertaken to explore variations in experiences and so a large sample was required. Participants gave detailed in-depth narrative accounts of their experiences. |
