# Supplementary material for: What outcomes are important to families with a lived experience of stillbirth? A qualitative study to inform the development of a core outcome set for stillbirth care
Source: PLoS One. 2026 May 19;21(5):e0347544. doi: 10.1371/journal.pone.0347544 (PMC13186333; doi:10.1371/journal.pone.0347544)
Supplement: S2 File — (DOCX) [file pone.0347544.s002.docx]

Supporting information 2: Topic Guide

1. The parent experience

**I’d like you to tell me your story with as much detail as possible. Then I may have some extra questions if you have not covered them already. We would like to find out your experience and we are particularly interested in how your loss has affected you/and your partner.**

1. **The diagnosis of the stillbirth**

- Can you take me back to the beginning of you story, perhaps start from when you first found out you were pregnant?

1. **The time between diagnosis and birth (If applicable)**

- What happened after you found out you your baby had died e.g. did you go home/stay in hospital/be induced?

1. **The birth of your baby**

- What was your experience of giving birth to your baby?
- How do you feel about the way you gave birth now?

1. **Your stay in hospital**
   - - What happened after your baby was born?
2. **Memory Making**
   - How did you spend time with your baby? If you were unable to spend time with baby, then why not?

*Examples include: did you see or hold the baby, did you take photos, foot prints*

- How did it make you feel at the time?
- What do you feel about the experience now?
- Which memories are the most meaningful to you now?

1. **Post-mortem & hospital tests**

- What hospital tests did you or your baby have afterwards?
- What information did you learn from the post-mortem and/or additional tests?
- How did you feel about your choice at the time & how do you feel about it now? (If did or did not have PM)

1. **The review process by the hospital (perinatal mortality review)**

- What was your experience of the hospital review process of you and your baby’s case?
- Were the involved in the case?
- If parental engagement in review – how did it affect them?

1. **The funeral**

- What information & support were you given about the baby’s funeral?
- If had funeral, can you tell me more about it
- How did that make you feel at the time/how do you feel about it now?

1. **When you went home**

- Can you tell me about how you made the decision about going home?
- What happened in the days/weeks after you went home?
- How did you feel when you went home?

1. **Follow up care by healthcare professionals (e.g hospital consultant, midwife, GP, anyone else?)**

- What follow up care did you receive from the hospital e.g. follow up appointment with consultant, bereavement midwife?
- What advice were you given in your follow up appointment about becoming pregnant again?
  - *Were you told how long you should wait before becoming pregnant again (if not discussed)? What will the care be like? What will the birth be like? What were your thoughts about this?*
- What was the contact with the GP/community midwife like?
- What was the impact of the healthcare professionals care on you?

1. **Additional care**

- Following your experience did you seek any further professional advice or care?
  - *Examples include second opinion, counselling*
- If yes, how did you feel about this care?
- What was the impact of counselling on you? May consider benefits and harms?
- Were there any other ways/methods you dealt with the stillbirth?
  - *Examples could include exercise, mindfulness, charity work, yoga, new hobby, support groups*

1. **Support groups**

- Did you seek any help from support groups?
- How did that impact you?
- Did you seek any support from online communities?

1. **Plans for future pregnancies**

- What were your thoughts about becoming pregnant again?

*What influenced your decision? Did you seek any alternative advice? What were the sources of your information?*

1. **Outcomes**

**1a) Impact on next pregnancy (if applicable)**

- Have you had any more pregnancies since? Tell me about your next pregnancy
- How did your previous experience affect your next pregnancy?
- How were you looked after in your next pregnancy?
- Examples: were you treated differently by medical professionals during antenatal care? did you have any extra care, appointments, scans or tests? Was the type of birth different?
- How did becoming pregnant affect you and/or your partner?
- How did being pregnant affect your health? (physical & mental)
- What support (psychological) was offered during pregnancy and after birth?
- Were there any complications in the subsequent pregnancy?
- Did the subsequent pregnancy have an effect on other areas of your life?

**1b) If no further pregnancies:**

- How did not becoming pregnant again affect you?
- Did you seek any fertility treatment & If yes, could you tell me about it and how did it effect you?

**2) Impact of experience on physical & mental health**

**Opening statement: In this part of interview I will ask about you and your partners health including the impact the stillbirth had on your physical and mental health**

- How do you think your experience affected your health in the short term (physical and mental)?
- How do you think your experience affected your health in the long term (physical and mental)?

If does not mention:

Has your experience affected your mental health?

Have you had low mood/anxiety/PTSD?

How has your experience affected your physical health?

Has is it affected your sleep?

Has your experience affected your body image/self-esteem?

- Did you do anything to improve your health after the experience? What were you encouraged to do?
- Could there have been anything done to minimise the impact on you and your health?
- Have any new medical conditions emerged since the stillbirth? Have you had any treatment/therapy?
- What medications have you taken (if any) following the stillbirth?

1. **Relationships**

- How has your experience affected your relationships?
  - Your partner (weeks, months, years); Your family (weeks, months, years); Your friends (weeks, months, years)
- What was the impact of new babies on you?

1. **Relationship with children (if applicable)**
   - How has your experience affected your relationship with your children?
   - Has your experience affected bonding/parenting with your children?
   - Have your children needed to have any additional support?
2. **Communication**

- How did you tell people about your stillbirth?
- How did they respond and how did it affect you?
- What was the impact of social media/media/news stories on you?

1. **Employment - going back to work/Finances**

- How did you break the news to your colleagues?
- How did your experience affect your job? (if applicable)
- How did the response of your employer affect you (and/or your decision to return to work)?
- How did your experience affect your finances? How did finances affect your decision making?
- If money was no issue, when would have been the right time to return to work in your opinion?

1. **Outcomes to develop core outcome set**

- If we were going to improve care or research after stillbirth what would be important in your opinion to measure to see if the care worked? Examples include mental health, physical, return to work
- If we were going to improve health after stillbirth what aspect of health would be important to you to improve?
- If unable to answer: How would you improve about the care you received. If that care was improved how would that affected your life and health?

1. **Key messages to parents & healthcare professionals**

**Opening statement: Bearing In mind parents might receive this information whilst in hospital/preparing to go into hospital or newly discharged…**

- What would be your advice to parent who might be going through a similar experience?
- What would be your overall key messages to healthcare professionals looking after parents who experience stillbirth?
