# Supplementary material for: What outcomes are important to families with a lived experience of stillbirth? A qualitative study to inform the development of a core outcome set for stillbirth care
Source: PLoS One. 2026 May 19;21(5):e0347544. doi: 10.1371/journal.pone.0347544 (PMC13186333; doi:10.1371/journal.pone.0347544)
Supplement: S4 Table — (DOCX) [file pone.0347544.s004.docx]

**Table 2: Strategies adopted in study to increase trustworthiness and rigor**

|  | **Strategies adopted in study to increase trustworthiness and quality** |
| --- | --- |
| **Sensitivity to context** | Systematic review conducted on outcomes reported in stillbirth care research to inform topic guide  Awareness of previously conducted qualitative research on stillbirth care research  Establishment of parent involvement group and consultation with parents throughout the study to inform study design, recruitment and analysis  Engagement with key stakeholders (e.g., charity support groups) to help recruit participants  Researchers experienced and sensitive to ethical issues of conducting qualitative research with parents who have experienced stillbirth  Prior qualitative research with bereaved parents conducted by research team |
| **Commitment and rigour** | Line by line independent and duplicate coding to extract outcomes  Three levels of coding utilised  Attention to maximum variation in recruitment sample  Consideration deviant views in analysis  Large number of verbatim quotes to justify findings  Supervision by an experienced qualitative researcher (LH) |
| **Transparency and coherence** | Thorough analysis and extensive write up  Coding tree provided and supplementary quotes available  Thematic concepts coherent with wider qualitative research  Reporting of the lived experience using quotes  Reflexivity demonstrated by researcher |
| **Impact and importance** | Addresses research gap on what outcomes are important to measure for stillbirth care research  Will inform the development of a core outcome set for stillbirth care research which will in turn improve evidence synthesis and strengthen the evidence on what care is best for parents after stillbirth |
