# Supplementary material for: What outcomes are important to families with a lived experience of stillbirth? A qualitative study to inform the development of a core outcome set for stillbirth care
Source: PLoS One. 2026 May 19;21(5):e0347544. doi: 10.1371/journal.pone.0347544 (PMC13186333; doi:10.1371/journal.pone.0347544)
Supplement: S5 Table — (DOCX) [file pone.0347544.s005.docx]

*Outcomes highlighted in yellow reported by previous stillbirth care studies as reported by Bakhbakhi D, Siassakos D, Davies A, Merriel A, Barnard K, Stead E, et al. Interventions, outcomes and outcome measurement instruments in stillbirth care research: A systematic review to inform the development of a core outcome set. *BJOG*. 2023; 130(6): 560–576. <https://doi.org/10.1111/1471-0528.17390>

| **Clinical care outcomes** | |
| --- | --- |
| **Diagnosis, labour and birth** | |
| **11 codes** | **8 outcomes** |
| Emergency caesarean section | Emergency caesarean section |
| Diagnosis of stillbirth | Experience of how the stillbirth was diagnosed |
| Time between diagnosis and birth | Length of time from the identification of stillbirth to the birth |
| Remembering the experience | Memory of the birth experience |
| Good memory |  |
| Mode of birth | Mode of birth |
| Labour and birth | Experience of birth and labour |
| Pain and analgesia | Use of analgesia during labour and birth |
| Wanting clarity of the event following analgesia | Side effects of treatment |
| Side effects of analgesia |  |
| **Investigations** | |
| **27 codes** | **21 outcomes** |
| Identification of Down's syndrome on PM | Identification of genetic abnormality in baby (including congenital anomaly) |
| Fetal heart abnormality |  |
| Disagreement with healthcare professional | Disagreement with healthcare professionals about cause of death |
| Explanation for cause of death | Identification of cause of death |
| No explanation for cause of death |  |
| Searching for clues |  |
| Uncertainty about cause of death |  |
| Feeling more prepared in next pregnancy | Feeling more prepared in next pregnancy |
| Antiphospholipid syndrome | Identification of anti-phospholipid syndrome |
| Identification of Streptococcus B as cause | Identification of infection as cause of stillbirth |
| Type 2 diabetes | Identification of type 2 diabetes |
| Change things for the future | Impact of post-mortem on the future |
| Improved antenatal education | Improved antenatal information |
| Stillbirth happening to other people | Parent's realising stillbirth happens to other people |
| Knowledge and understanding | Knowledge and understanding of stillbirth |
| Hindsight | Doing things differently in hindsight |
| Parental involvement | Parental engagement in the perinatal mortality review process |
| Review process | Perinatal mortality review process conducted |
| Post-mortem and investigations | Post-mortem and investigations conducted |
| Preventing stillbirth | Prevent stillbirth recurrence |
| Prevention of future stillbirth |  |
| Second opinion | Second opinion sought to review care |
| Delay in results | Length of time it took for post-mortem results |
| Long wait for post-mortem results |  |
| Parent's understanding of cause | Parent's understanding of cause of death |
| Risk of recurrence discussed | Risk of recurrence of stillbirth |
| Feelings about post-mortem | Feelings about post-mortem |
| **Postpartum** | |
| **26 codes** | **17 outcomes** |
| Adverse events or effects | Adverse events |
| Lactation suppression | Lactation management |
| Milk |  |
| Need to go to theatre to control bleeding | Need to go to theatre to control bleeding |
| Survival or survived | Parental survival |
| Physical postpartum impact | Physical postpartum impact |
| Physically and emotionally exhausting |  |
| Postpartum anaemia | Postpartum anaemia |
| Postpartum haemorrhage | Postpartum haemorrhage |
| Postpartum body and emotional changes | Postpartum body and emotional changes |
| Postpartum complications | Postpartum complications |
| Postpartum infection | Postpartum infection |
| Renal and urinary |  |
| Postnatal healing | Postpartum recovery |
| Postnatal recovery |  |
| Retained placenta | Retained placenta |
| Vomiting | Postpartum vomiting |
| Physically exhausting | Physical functioning |
| Physical functioning |  |
| Physical impact |  |
| Being able to function |  |
| Impact on weight | Impact on weight |
| Lost weight |  |
| Weight gain |  |
| More exercise | More exercise |
| Reduced physical activity | Impact on physical activity |
| **Care experience** | |
| **101 codes** | **32 outcomes** |
| Appropriate care | Appropriate care provided |
| Complaint | Complaint made by parents |
| Continuity of care | Continuity of care |
| Handover of care |  |
| Referral of services |  |
| Transfer of care |  |
| Having enough space for parents | Dedicated space for parents |
| Delivery of care | Experience of care |
| Aspects of care |  |
| Feeling abandoned |  |
| Feeling let down or disappointed |  |
| Feeling of safety |  |
| Feeling prepared |  |
| Feeling rushed |  |
| Offering care |  |
| Humiliating |  |
| Impact of lack of care |  |
| Impersonal care |  |
| Being taken seriously |  |
| Inappropriate care |  |
| Individual care |  |
| Lack of care |  |
| Lost faith in care |  |
| Making the experience normal for parents |  |
| Managed to do small things |  |
| More human care |  |
| Negative care |  |
| Being forgotten about |  |
| Incorrect care |  |
| Negative care from other professionals |  |
| Negative care from healthcare professionals |  |
| Apathy from healthcare professionals |  |
| Positive care |  |
| Privacy |  |
| Re-offering care |  |
| Staff show that they care |  |
| Transparent care |  |
| Communication | Experience of communication |
| Being listened to |  |
| Empathy |  |
| Feeling informed or not |  |
| Having the correct information |  |
| Having consistent information |  |
| Incorrect information |  |
| Involved in process of discussion | Shared decision making |
| Making memories and spending time with baby | Given opportunities to make memories and spend time with baby |
| Barriers to making memories |  |
| Parents feeling they could do as much as they could for baby |  |
| Experienced healthcare professional | Having an experienced healthcare professional |
| Having autonomy | Having choices, options or knowing what parents are allowed to do |
| Having choices, options or what parents are allowed to do |  |
| Having space to think about decisions | Having space to think about decisions |
| Impact on healthcare professionals | Impact on healthcare professionals |
| Improvements to care | Improvements to care from feedback |
| Getting feedback from parents |  |
| Key messages to healthcare professionals |  |
| Involving the whole family | Including family and friends in memory making |
| Including father | Including the partner in the bereavement care |
| Treating things on an individual basis | Individualised care |
| Ideal timing of care | Information given in timely manner |
| Litigation and negligent care | Litigation and negligent care |
| Problems with monitoring baby leading to litigation |  |
| Taking responsibility |  |
| Follow up | Postnatal emotional and/or physical health check conducted |
| Emotional and mental check up |  |
| Physical check up |  |
| Postnatal check |  |
| Lack of follow up |  |
| Leaving baby | Given information about going home and leaving baby |
| Stillbirth certificate |  |
| Not knowing what is normal or not | Given or provided with information about what to expect |
| Registering death | Provided medical certificate certifying stillbirth and information about registering the death. |
| Respect | Respectful care |
| Root cause analysis | Root cause analysis conducted |
| Feel like healthcare professional was making an effort to find answer | Satisfaction with care |
| Feeling anonymous |  |
| Finding help for yourself | Self-referral for help |
| Self-help and care |  |
| Self-refer |  |
| Being able to ask for help |  |
| Baptism | Spiritual support |
| Chaplain |  |
| Support from healthcare professional | Support from healthcare professionals |
| Feeling supported |  |
| Healthcare professionals |  |
| Bereavement midwife |  |
| Community midwife |  |
| GP |  |
| Interaction with healthcare & allied professionals |  |
| Healthcare professional did not dismiss anything |  |
| Occupational health |  |
| Help answering questions |  |
| No outside support |  |
| Point of contact |  |
| Feeling calm |  |
| Accessibility to support |  |
| Reassurance |  |
| Training of staff | Training of staff |
| Trust | Trust |
| Readmission/attendance at hospital | Readmission or attendance to hospital |
| Unscheduled hospital visits in subsequent pregnancy | Unscheduled hospital visits in a subsequent pregnancy |
| **Outcomes when a stillbirth occurs in a multiple pregnancy** | |
| **42 codes** | **41 outcomes** |
| Twin pregnancy still twin pregnancy following stillbirth | Acknowledgement of multiple pregnancy |
| Admission to neonatal intensive care unit | Admission to neonatal intensive care unit |
| Antenatal baby complications | Antenatal baby complications in a multiple pregnancy |
| Bereavement care multiple pregnancy | Bereavement care multiple pregnancy |
| Birth outcomes subsequent pregnancy | Birth outcomes in multiple pregnancy |
| Don't blame multiple sibling | Blame surviving multiple |
| Care experience in a multiple pregnancy | Care experience in a multiple pregnancy |
| Bringing the multiples together |  |
| Concern about developmental milestones of surviving multiple | Concern about developmental milestones of surviving multiple |
| Concern about health of surviving multiple | Concern about health of surviving multiple |
| Surviving multiple might die | Concern about survival of multiple |
| Conservative management of multiple pregnancy | Conservative management of multiple pregnancy |
| Delayed grief in multiple pregnancy | Delayed grief in multiple pregnancy |
| Disagreement in management | Disagreement in management |
| Feeding surviving multiple | Feeding surviving multiple |
| Grief in a multiple pregnancy | Grief in a multiple pregnancy |
| Concentrating on surviving multiple | Concentrating on surviving multiple |
| High APGAR score surviving multiple | High APGAR score surviving multiple |
| Infection of surviving multiple | Infection of surviving multiple |
| Jaundice of surviving multiple | Jaundice of surviving multiple |
| Live birth of surviving multiple | Live birth of surviving multiple |
| Medication to stop premature delivery | Medication to stop premature delivery |
| Mode of birth in discussed in multiple pregnancy | Mode of birth in discussed in multiple pregnancy |
| Monitoring of multiple pregnancy | Monitoring of multiple pregnancy |
| Necrotising enterocolitis in surviving multiple | Necrotising enterocolitis in surviving multiple |
| Neonatal death of surviving multiple | Neonatal death of surviving multiple |
| Neonatal outcomes surviving multiple | Neonatal outcomes surviving multiple |
| Perspective of surviving multiple sibling | Perspective of surviving multiple sibling |
| Premature delivery of multiple pregnancy | Premature delivery of multiple pregnancy |
| Premature labour of multiple pregnancy | Premature labour of multiple pregnancy |
| Psychological impact of surviving multiple | Psychological impact of surviving multiple |
| Psychosocial support outcomes multiple | Psychosocial support outcomes multiple |
| Reduced fetal movements | Reduced fetal movements |
| Relief when surviving multiple born | Relief when surviving multiple born |
| Sepsis of surviving multiple | Sepsis of surviving multiple |
| Steroid injections for multiple pregnancy | Steroid injections for multiple pregnancy |
| Support for surviving multiple | Support for surviving multiple |
| Survival outcomes in multiple pregnancy | Survival outcomes in multiple pregnancy |
| Surviving multiple might have brain damage | Surviving multiple might have brain damage |
| Surviving multiple prematurity | Surviving multiple prematurity |
| Uncertainty about outcome of surviving multiple | Uncertainty about outcome of surviving multiple |
| Weight of surviving multiple | Weight of surviving multiple |
| **Grief and psychological** | |
| **Grief and bereavement** | |
| **90 codes** | **25 outcomes** |
| Move forwards | Acceptance of grief |
| Move on with life |  |
| Not affected moving forwards with lives |  |
| Trying to look forwards |  |
| Acceptance |  |
| Making the experience feel real |  |
| Realisation |  |
| Most important thing that has happened | Acknowledging the baby |
| Acknowledging loss |  |
| Baby will always be important |  |
| Focus on baby |  |
| Identity of baby or real baby |  |
| Acknowledging loss | Acknowledging loss |
| Appearance of baby | Appearance of baby |
| Care of the baby's body | Care and respecting body of baby |
| Funeral and burial or cremation of baby | Coping with funeral and funeral arrangements |
| Ability to deal with the funeral |  |
| Location of grave |  |
| Time between birth and funeral |  |
| Emotionally detach | Coping with grief |
| Trying to forget |  |
| Coping |  |
| Dealing with practicalities |  |
| Focusing attention on something positive |  |
| Keeping busy |  |
| Life doesn't stop when you've lost your baby |  |
| Making sure there was something left in life |  |
| Pets |  |
| Culture |  |
| Flowers |  |
| Differences between men and women |  |
| Forgetting the experience |  |
| Know how to deal with own grief |  |
| Knowing how to deal with things |  |
| Triggering events |  |
| Trying to forget |  |
| Anger | Feelings of grief |
| Bitter with the world |  |
| Dealing with grief individually |  |
| Denial |  |
| Despair |  |
| Feeling lost |  |
| Feeling shocked |  |
| Having nothing |  |
| Healing process |  |
| Immediate reactions of parents |  |
| Loss of hopes and dreams |  |
| Plans falling through/everything falling apart |  |
| Surreal |  |
| Grief and bereavement outcomes | Grief |
| Grief |  |
| Impact on grandparents | Impact of grief on grandparents |
| Impact on sibling | Impact of grief on siblings |
| Change in physical behaviour |  |
| Deal with parent's grief to enable sibling to grieve | Impact of grief on whole family |
| Father's perspective |  |
| Including father |  |
| Involving the whole family |  |
| Talking about loss | Perceived opportunities to talk about stillbirth or loss |
| Taboo subject |  |
| Telling people |  |
| Concern about life or death | Concern about life or death |
| Difficulty grieving | Difficulty grieving |
| Not wanting body to go back to normal | Physical grief |
| Physical grief |  |
| Mental affected the physical |  |
| Locking things down | Processing grief |
| No opportunity to deal with this properly |  |
| No time to process grief |  |
| Not given the opportunity to grieve |  |
| Not processing event properly |  |
| Process things |  |
| Processing event |  |
| Still processing things |  |
| Parenthood or feeling like baby is theirs | Recognition of parenthood |
| Maternal instinct |  |
| Always part of lives | Remembering the baby, coping with key milestones, anniversaries |
| Legacy of baby |  |
| Remembering baby |  |
| Milestones, anniversaries, religious festivals |  |
| Self-blame, guilt & failure | Self-blame guilt and failure |
| Impact on grief journey | Support with life-long grief |
| Life long |  |
| Understanding somebody's grief | Understanding grief |
| Unrecognised grief | Unrecognised grief |
| Delayed grief | Unresolved grief |
| Delayed reaction |  |
| Valuing loss and respecting baby | Valuing loss and respecting baby |
| Valuing loss same as live baby |  |
| Wanting this baby |  |
| **Mental health** | |
| **27 codes** | **13 outcomes** |
| Alcohol use | Alcohol and drug use |
| Drank more alcohol |  |
| Feeling anxious | Anxiety |
| Psychological support | Counselling or psychological support |
| Counselling |  |
| Opportunity to offload or sounding board |  |
| Lose your way in life | Depression |
| Difficulty concentrating | Difficulty concentrating |
| Difficulty with memory | Difficulty with memory |
| Eating disorder | Eating disorder |
| Mental health | Impact on mental health |
| Mental health impact |  |
| Damaging |  |
| Did harm |  |
| Difficulty planning ahead |  |
| Breakdown | Mental breakdown |
| Wellbeing of partner | Mental health of partner |
| Need for further intervention or counselling | Need for mental health intervention |
| Treatment or therapy |  |
| Timing of treatment or therapy |  |
| Bad memory | Post-traumatic stress disorder or trauma |
| Feeling horrified or horrific |  |
| Flashbacks |  |
| PTSD |  |
| Traumatic or Trauma |  |
| Lose the value of your own life | Suicidal thoughts |
| Suicidal thoughts |  |
| **Emotional health** | |
| **51 codes** | **18 outcomes** |
| Not processing emotion | Ability to process emotions |
| Emotional support | Access to emotional support |
| Self-image | Body image |
| Not wanting body to go back to normal |  |
| Confidence | Confidence |
| Emotional functioning | Emotional functioning |
| Impact of unanswered questions | Emotional wellbeing |
| Distressing or upsetting |  |
| Mixture of emotions |  |
| Negative thoughts |  |
| Wellbeing |  |
| Empowerment | Empowerment and control |
| Parental control |  |
| Powerless |  |
| Fear | Fear |
| Fear of death | Fear of death |
| Fear of hospitals | Fear of hospitals |
| Feeling jealous of other pregnant women | Feeling jealous |
| Shame | Feeling shame or embarrassment |
| Embarrassment |  |
| Hiding baby |  |
| Not wanting attention as still appear pregnant |  |
| Feeling more cynical | Negative emotions |
| Feeling paranoid |  |
| Feeling resentment |  |
| Feeling terrified |  |
| Frightened |  |
| Lose perspective |  |
| Scared |  |
| Self-destruction |  |
| Unable to think about future |  |
| Questioning this world |  |
| Forgetting the experience |  |
| Feeling happy | Positive emotions |
| Having hope |  |
| Optimism |  |
| Pride |  |
| Think positively |  |
| No regret | Regret |
| Regret |  |
| Strength of mother | Resilience |
| Resilience |  |
| Feeling worthless | Self-esteem |
| Low self esteem |  |
| Self-doubt |  |
| Self-esteem |  |
| Useless |  |
| Physically and emotionally exhausting | Stress |
| Unable to relax |  |
| Mentally exhausting |  |
| Stress |  |
| **Whole person outcomes** | |
| **37 codes** | **18 outcomes** |
| Life changing | Change in life |
| Changes life forever |  |
| Affects every area of life | Quality of life |
| Quality of life |  |
| Global quality of life |  |
| Feel more normal | Change in normality |
| Feeling abnormal |  |
| Never going to be same again |  |
| Something missing |  |
| Changed as a person | Changed as a person |
| Concern about health and fitness | Concern with health and fitness |
| General health | General health |
| Help others | Help others who have experienced stillbirth |
| Spirituality | Impact on spirituality |
| Impact on health in the future | Long term future health |
| Long term impact |  |
| Measure outcomes at different points | Long term outcomes |
| New medical diagnosis | New medical diagnosis |
| New normal | New normal |
| Have scientific meaning | Participating in research |
| Perceived health status | Perceived health status |
| Feeling grateful | Feeling gratitude |
| Positive or personal growth outcomes | Positive impact or post traumatic growth |
| Less naive |  |
| Positive impact |  |
| Being more self-aware |  |
| Better person |  |
| More empathetic |  |
| No longer worried about futile things |  |
| Not expecting too much of oneself |  |
| See things from a different perspective |  |
| Turning something negative into something positive |  |
| Got back to myself | Return to normality |
| Returning to normality |  |
| To get back to where we were |  |
| Family normal |  |
| Sleep | Impact on sleep |
| **Social and family** | |
| **Social outcomes** | |
| **55 codes** | **26 outcomes** |
| Agoraphobia | Agoraphobia |
| Isolation and feeling alone or lonely | Isolation or feeling alone |
| Protecting others |  |
| Family | Impact on relationship with family |
| Helped keep relationships | Impact on relationships with friends |
| Loss of friendships |  |
| New friendships |  |
| Sharing with friends or not |  |
| Support | Perceived support from others |
| Unhelpful social support | Satisfaction with social support |
| Social functioning | Social functioning |
| Seeing or hearing other children, babies or pregnant women |  |
| Helped me get out of the house |  |
| Seeing or hearing other children, babies or pregnant women |  |
| Social interaction | Social impact |
| Avoidance |  |
| Difficulty being in social situations |  |
| Found small talk difficult |  |
| Help with social situations |  |
| Misinterpreting other people's responses |  |
| No social life |  |
| Media | Social media support used |
| Media |  |
| Social phobia | Social phobia |
| Stigma | Stigma |
| Not feeling judged |  |
| Support books | Support books or leaflets used |
| Supporting family members | Support for family members |
| Support from family | Support from family |
| Friends | Support from friends |
| Support from others who have experienced loss | Support from others who have experienced loss |
| Support groups | Support groups attended |
| Support resources | Support resources used |
| Wanting to leave the UK | Wanting to leave the country |
| Leave country |  |
| Difficulty being creative | Impact on job role |
| Impact on job role |  |
| Work |  |
| Getting on with life tasks | Impact on role in life |
| Have to get on with life |  |
| Role functioning |  |
| Being able to work | Impact on work |
| Energy levels needed for work |  |
| Help with practical aspects of work |  |
| Taking time off work |  |
| Change in career | Changed job |
| Additional paternity leave | Requiring parental leave |
| Maternity leave or paternity leave |  |
| Care that helped return to work | Support with returning to work |
| Employer not aware of maternity or paternity leave rights |  |
| Phased return |  |
| Reactions from colleagues |  |
| Support with returning to work |  |
| Working from home |  |
| Lost job | Unemployment |
| **Partner and family relationship outcomes** | |
| **34 codes** | **21 outcomes** |
| Counselling helping relationship | Counselling helping relationship |
| Couple support | Couple support needed |
| Delayed grief in partner | Delayed grief in partner |
| Emotional health of partner | Emotional health of partner |
| Father needing support | Father needing support |
| Grieving differently | Grieving differently from partner |
| Impact on father | Impact on father |
| Relationship with partner | Impact on relationship with partner |
| Affects every relationship |  |
| Becoming pregnant again to try and help the relationship |  |
| Brought closer together |  |
| Closer together and further apart |  |
| Got married afterwards |  |
| Prevent relationship breakup |  |
| Relationship with partner outcomes |  |
| Relationships |  |
| Impact on subsequent relationship | Impact on subsequent relationship |
| Mental health of partner | Mental health of partner |
| Needing counselling for relationship difficulties | Needing counselling for relationship difficulties |
| Negative impact on relationship | Negative impact on relationship |
| No long-term effects on relationship | No long-term effects on relationship |
| Rituals to remember baby together | Rituals to remember baby together |
| Social impact on father | Social impact on father |
| Mother looking after partner | Support for the father |
| Need to consider partner as well |  |
| Not being able to support mother |  |
| Father support | Support from father |
| Partner being supportive |  |
| Partner not being supportive |  |
| Father looking after mother |  |
| Supporting each other | Supporting each other |
| Trying to get pregnant again affecting the relationship | Trying to get pregnant again affecting the relationship |
| Unable to support each other | Unable to support each other |
| Wellbeing of partner | Wellbeing of partner |
| **Outcomes related to older children** | |
| **66 codes** | **10 outcomes** |
| Attachment and bonding with baby | Attachment and bonding with children |
| Child continuing to talk about her | Grief of existing children |
| Children remembering baby |  |
| Children talking about stillbirth |  |
| Confusion |  |
| Deal with parent's grief to enable sibling to grieve |  |
| Education | Impact on children's behaviour |
| Change in children's behaviour |  |
| Change in physical behaviour |  |
| Children and Siblings | Impact on existing children |
| No impact on children |  |
| Child anxiety about medical problems | Impact on mental health of child |
| Child aware of parents being upset |  |
| Child separation anxiety |  |
| Child talking to stranger |  |
| Child upset |  |
| Child wanting sibling |  |
| Children suffering |  |
| Children upset due to parents upset |  |
| Children's life different |  |
| Traumatic |  |
| Better parent | Impact on parenting |
| Children and parenting |  |
| Concentrating on surviving children |  |
| Conflicting priorities spending time with baby vs child |  |
| Difficulty concentrating on existing child |  |
| Kept going due to child |  |
| Long term anxiety about existing children |  |
| Maternity leave to focus on child |  |
| More anxious parenting |  |
| More cautious |  |
| More patient |  |
| No impact on parenting |  |
| No support for parenting |  |
| Over-protective |  |
| Separation anxiety |  |
| Try to be normal for child |  |
| Worse parent |  |
| Impact on sibling | Impact on sibling |
| Long term impact on sibling |  |
| Child not meeting baby | Including existing child in memory making |
| Children meeting baby |  |
| Regret child not meeting baby |  |
| Physical or verbal abuse | Physical or verbal abuse |
| Scapegoat |  |
| Advice from bereavement counsellor | Support for existing children |
| Advice from hospital |  |
| Asking more questions as gets existing |  |
| Charity support and advice on what to say |  |
| Child counselling through school |  |
| Child seeing psychologist |  |
| Difficulty explaining |  |
| Each child different |  |
| Important children aware |  |
| No support on telling children |  |
| Not understanding |  |
| Parenting support |  |
| Providing explanation of what happened |  |
| School |  |
| Support books for children |  |
| Support for existing children |  |
| Support for parenting |  |
| Support from school |  |
| Support important for parents |  |
| Support with siblings |  |
| Telling children about stillbirth |  |
| **Economic** | |
| **1 code** | **1 outcome** |
| Impact on finances | Financial cost of stillbirth to parents |
| **Future pregnancy and children** | |
| **Planning subsequent pregnancy** | |
| **17 codes** | **7 outcomes** |
| Partner vasectomy | Contraception |
| Fertility decisions | Fertility treatment or support |
| Fertility investigations |  |
| Fertility treatment |  |
| Help to become pregnant again |  |
| Reassurances about fertility |  |
| Support with next pregnancy and infertility |  |
| Trying to become pregnant again |  |
| Timing of birth of next pregnancy | Inter-pregnancy interval |
| Timing of birth of next pregnancy |  |
| Timing of next pregnancy |  |
| Information about future pregnancies | Pre-conception counselling |
| Measures to reduce risk of subsequent stillbirth |  |
| Support with next pregnancy and infertility | Support with next pregnancy and fertility |
| Wanting another baby | Wanting another baby |
| Wanted to be pregnant again | Wanting to become pregnant again or not |
| Wanting another baby |  |
| **Subsequent pregnancy** | |
| **105 codes** | **84 outcomes** |
| Access to out of hours | Out of hour appointments |
| Access to specialised service in next pregnancy | Specialised antenatal care in a subsequent pregnancy |
| Management of future pregnancy |  |
| Additional appointments | Additional appointments |
| Additional midwife appointments | Additional midwife appointments |
| Additional scans in a subsequent pregnancy | Additional scans in a subsequent pregnancy |
| Additional testing in a subsequent pregnancy | Additional testing in a subsequent pregnancy |
| Antenatal class in a subsequent pregnancy | Antenatal class in a subsequent pregnancy |
| Anxiety in a pregnancy following a live birth | Anxiety in a subsequent pregnancy |
| Anxiety in a subsequent pregnancy |  |
| Aspirin in subsequent pregnancy | Treatment in a subsequent pregnancy |
| Decompression |  |
| Attachment to baby in a subsequent pregnancy | Attachment to baby in a subsequent pregnancy |
| Baby complications in a subsequent pregnancy | Baby complications in a subsequent pregnancy |
| No complications of baby in a subsequent pregnancy |  |
| Baby NICU in subsequent pregnancy | Baby NICU in subsequent pregnancy |
| Becoming pregnant again having negative impact on relationship | Impact on relationship in a subsequent pregnancy |
| Being careful in next pregnancy | More careful in a subsequent pregnancy |
| Bereavement support in a subsequent pregnancy | Bereavement support in a subsequent pregnancy |
| Breastfeeding in a subsequent pregnancy | Breastfeeding support in a subsequent pregnancy |
| C-section in next pregnancy | Caesarean section in a subsequent pregnancy |
| Care that does not recognise subsequent pregnancy | Care that acknowledges previous stillbirth |
| Tried to treat next pregnancy as normal |  |
| Care that helped with mental health | Mental health care in a subsequent pregnancy |
| Care that recognises subsequent pregnancy | Care that recognises subsequent pregnancy |
| Choice in mode of birth | Choice in mode of birth in a subsequent pregnancy |
| Chronic kidney disease in a subsequent pregnancy | Chronic kidney disease in a subsequent pregnancy |
| Concern about health of next baby | Concern about survival of subsequent child |
| Concern whether next baby will survive |  |
| Guarantee baby will arrive safely in next pregnancy |  |
| Congenital heart defect in subsequent pregnancy | Congenital heart defect in subsequent pregnancy |
| Continuity in a subsequent pregnancy | Continuity in a subsequent pregnancy |
| No continuity of care in a subsequent pregnancy |  |
| Coping in a subsequent pregnancy | Coping in a subsequent pregnancy |
| Counsellor in a subsequent pregnancy | Counsellor in a subsequent pregnancy |
| Denial of pregnancy | Denial of pregnancy |
| Depression in a subsequent pregnancy | Depression in a subsequent pregnancy |
| Discussion on mode of birth | Discussion on mode of birth in a subsequent pregnancy |
| Emotional fatigue of subsequent pregnancy | Emotional impact on subsequent pregnancy |
| Emotional impact on next pregnancy |  |
| Feeling brave in next pregnancy |  |
| Feeling shame about stillbirth in a subsequent pregnancy |  |
| Mixture of emotions |  |
| No benchmark or milestones will be nervous until baby born |  |
| Experience of care in a subsequent pregnancy | Experience of care in a subsequent pregnancy |
| Negative experience of care |  |
| Father worried as can't feel kicking | Father anxiety in a subsequent pregnancy |
| Fear | Fear in a subsequent pregnancy |
| Fear stillbirth will reoccur | Fear of stillbirth recurrence in a subsequent pregnancy |
| Feeling in control | Control in a subsequent pregnancy |
| No control in a subsequent pregnancy |  |
| Feeling more prepared in next pregnancy | Feeling more prepared in the next pregnancy |
| Helped with next pregnancy |  |
| Future healthy pregnancy and live birth | Future healthy pregnancy and live birth |
| Gestational diabetes | Gestational diabetes in a subsequent pregnancy |
| Grief in a subsequent pregnancy | Grief in a subsequent pregnancy |
| Growth restriction | Growth restriction in a subsequent pregnancy |
| Happiness when baby born | Happiness when baby born |
| Increased health service use in next pregnancy | Increased health service use in next pregnancy |
| Induction in next pregnancy | Induction of labour in next pregnancy |
| Jaundice in subsequent pregnancy | Jaundice of baby in a subsequent pregnancy |
| Less naive | Less naive in subsequent pregnancy |
| Location of birth in the next pregnancy | Decision of location of birth in the next pregnancy |
| Location of birth same as stillbirth |  |
| Maternal complications | Maternal complications in a subsequent pregnancy |
| Medical problems in a subsequent pregnancy | Medical problems in a subsequent pregnancy |
| Mental health in subsequent pregnancy | Mental health in subsequent pregnancy |
| Mental health support in a subsequent pregnancy | Mental health support in a subsequent pregnancy |
| Miscarriage in a subsequent pregnancy | Miscarriage in a subsequent pregnancy |
| Mode of birth in subsequent pregnancy | Mode of birth in a subsequent pregnancy |
| More difficult birth | Birth complications in a subsequent pregnancy |
| No reassurance until baby born | Concern about survival of baby in a subsequent pregnancy |
| Preoccupied with keeping next baby alive |  |
| Reassurance about heartbeat |  |
| Reassurance baby alive |  |
| No support from hospital in subsequent pregnancy | Support in a subsequent pregnancy |
| Obtaining a healthy pregnancy and live birth | Live birth in a subsequent in pregnancy |
| Overactive thyroid | Overactive thyroid in a subsequent pregnancy |
| Physical impact of subsequent pregnancy | Physical impact of subsequent pregnancy |
| Placental insufficiency in a subsequent pregnancy | Placental insufficiency in a subsequent pregnancy |
| Postnatal care in next pregnancy | Postnatal care and support in a subsequent pregnancy |
| Postnatal mental health after next baby | Postnatal mental health after next baby |
| Postnatal readmission in a subsequent pregnancy | Postnatal readmission in a subsequent pregnancy |
| Postpartum depression in subsequent pregnancy | Postpartum depression in subsequent pregnancy |
| Postpartum maternal complications | Postpartum maternal complications |
| Premature delivery next pregnancy | Pre-term birth in the subsequent pregnancy |
| Refusing medical treatment in a subsequent pregnancy | Refusing medical treatment in a subsequent pregnancy |
| Seeking additional care not provided by hospital | Seeking additional care not provided by hospital in a subsequent pregnancy |
| Social impact in a subsequent pregnancy | Social impact in a subsequent pregnancy |
| Specialist consultant in a subsequent pregnancy | Specialist consultant in a subsequent pregnancy |
| Stress in a subsequent pregnancy | Stress in a subsequent pregnancy |
| Subsequent pregnancy | Impact on subsequent pregnancy |
| Suicidal thoughts in a subsequent pregnancy | Suicidal thoughts in a subsequent pregnancy |
| Support groups in a subsequent pregnancy | Support groups in a subsequent pregnancy |
| Support team for subsequent pregnancy | Support team for subsequent pregnancy |
| Support with next pregnancy and infertility | Support with next pregnancy and infertility |
| Supportive care from professionals in a subsequent pregnancy | Supportive care from professionals in a subsequent pregnancy |
| Taking medication in next pregnancy | Taking medication in a subsequent pregnancy |
| Timing of birth of next pregnancy | Timing of birth of next pregnancy |
| Treatment in next pregnancy | Medical causes of stillbirth are addressed in a subsequent pregnancy |
| Trust in healthcare professionals in a subsequent pregnancy | Trust in healthcare professionals in a subsequent pregnancy |
| Unscheduled hospital visits in subsequent pregnancy | Unscheduled hospital visits in a subsequent pregnancy |
| Vaginal birth in a subsequent pregnancy | Vaginal birth in a subsequent pregnancy |
| Wanting a c-section in a subsequent pregnancy | Wanting a c-section in a subsequent pregnancy |
| **Subsequent Children** | |
| **10 codes** | **7 outcomes** |
| Attachment and bonding with baby | Attachment to subsequent children |
| Concern about health of next baby | Concern about health of next baby |
| Change in children's behaviour | Impact on child's behaviour |
| Impact on parenting subsequent children | Impact on parenting subsequent children |
| Subsequent children and siblings | Impact on subsequent children and siblings |
| Impact on younger sibling | Impact on younger sibling |
| No support needed | Support for subsequent children |
| Subsequent child remembering |  |
| Talking with subsequent child |  |
| Telling younger children |  |
