# Supplementary material for: What outcomes are important to families with a lived experience of stillbirth? A qualitative study to inform the development of a core outcome set for stillbirth care
Source: PLoS One. 2026 May 19;21(5):e0347544. doi: 10.1371/journal.pone.0347544 (PMC13186333; doi:10.1371/journal.pone.0347544)
Supplement: S6 File — (DOCX) [file pone.0347544.s006.docx]

**Clinical care outcomes**

**Diagnosis, labour and birth outcomes**

STB01: “Yeah, and then we just said, it was really weird like if it was a normal birth like when you watch One Born Every Minute and it was like go through the same thing then just the ending wasn’t good.”

STB02: “My first reaction was get him out, if he’s dead, get him out. The midwife explained in their best policy it was a case of they found that most parents needed the time and for future pregnancies it would be easier to do a natural birth.”

STB04 “… it is like being in a film of watching yourself and all of sudden the announcement that there wasn’t a heartbeat.”

STB05: “I do remember the doctor saying, ‘Don’t worry, we’ll get you through it with plenty of pain relief, you won’t even know’. I thought, ‘Oh right, okay’. We went in and it was a normal labour, there wasn’t any of this magic pain relief, they did try to give me morphine, which I don’t react well to morphine, it just makes me feel sick and lightheaded, it doesn’t actually give me any pain relief. Even if they had given me a paracetamol it would have been better than nothing.”

STB06: “When initially I was told I think if I’d been given a completely free choice I might have opted for a C-section, but I think that, in retrospect, that would have been the wrong choice because it probably would have made my subsequent pregnancies harder… so then when I was labouring with my other children, I wasn’t really having flashbacks to that particular – because they were quite different experiences.”

STB16: “I think the difficulty at that point is you start to wonder how does this work? Do they do a caesarean maybe to take this baby out or how does this baby come out of the tummy and arrive? For me, particularly, it was a bit of a shock to realise it was going to be a natural birth. I don’t know why I didn’t think that was going to be the case, but I suddenly felt that was a very odd way to do it, that there was an awful lot of work for [Mother’s name] to do for no reward at the end, I guess.”

STB17: “I think it was the right thing to do, not have a caesarean, I think definitely, for the reasons that they said; going through that process helps you prepare for what’s about to happen rather than just be put to sleep and it all be done for you. With regards to the actual birth it was the most horrendous thing I’ve ever done obviously, I don’t really remember much about it, I just remember being devastated really.”

STB17: “At that point for some reason I was obsessed with knowing that if I had another baby, I could have a caesarean, because I didn’t want to go through labour again, because my memory of labour was that and it was horrific. So he did me a letter to wherever I was having the baby that that would be okay.”

STB21: “‘Why can you not give me a C-section?’ and they said, ‘there is a risk that you die, it is very risky because your blood pressure is a risk for you, and we cannot afford that. If you want to get a C-section you need to sign that, but we will never recommend you do that’.”

STB21: “I was angry, I have to say, but today I am happy it was a natural birth. Unfortunately, you have all the pain, and you don’t keep the prize. These are my memories. This is something that just myself and her have got in common. No-one else can understand that.”

STB21: “Then I thought, why all this pain? Why all this hassle? Is it not necessary, so I said, ‘yeah I will go through all the drugs. I don’t want to feel anything. I don’t want to even feel when she is coming out, nothing. I don’t know what time it was. I think it was at night.”

STB14: “Um, it seemed a bit bizarre to me because they said, well you need to go home. So I still had my baby inside me, and they made me take all my maternity notes as well, which seemed pretty strange because you have to carry them round when you’re pregnant. They said, oh you have to take these as well, bring them back with you, and it just seemed really odd because I wasn’t, well, I’m not pregnant anymore. It was just little things like that were a bit annoying because I just thought they were a bit insensitive.”

**Postpartum medical outcomes**

STB28: “Unfortunately that first night I bled really heavily throughout the night and I definitely thought I was dying, myself actually, I couldn’t even speak I was so, I had lost maybe just so much blood.”

STB18: “ I think it would have been really helpful if I’d have had a bereavement midwife come here and see me for like an hour, you know, maybe a week or two after it happened and then kind of regularly for... even if it was for four weeks or six weeks, I think it would have been hugely helpful just to feel like... just to answer questions first of all because you’ve got so many questions in your mind; also to check you’re okay physically and mentally, you know, because you have been through a labour and then it’s sort of like, well because there’s no baby there it’s like nobody really cares. It’s weird, it’s like well... I mean I was lucky in that I didn’t have, you know, stitches or anything like that, but you could have, you could have physical things.

STB07: “There wasn’t like a six-week check-up, which I really assumed would have happened. Knowing that all mothers get that after giving birth”.

**Care experience outcomes**

STB19: “[Midwife’s name] our Midwife who delivered [Baby’s name] had been absolutely phenomenal, couldn't fault her at all. She even came back in, not the next night but the night after, she said, 'I tried to come up to you' she said, 'but you were fast asleep' she said, 'so I've come up tonight.' And like she sat there, she held my hand, she said, I went home, I had a cry, [tearfully] she said I was really upset for you and that really struck a chord with me.”

STB22: “If you’ve got all of the information, as a parent, you can then make up your own mind as to what you want to do and how you feel about things. It’s about having as much information as possible.”

STB27: “I think a way of monitoring that there is improvement, is if parents felt after the event that they were prepared, they were given the information required, they felt like they were part of the process of discussion.”

STB09: “They don’t really give you a choice. But I understand why. But they said, ‘You need to hold your baby…’ and all that and I think they were saying their study has shown, we found out afterwards that’s what they advise from a longer-term point of view. We didn’t really feel that we had a choice.”

STB20: “I remember my midwife had given me a paper that had options on it, my midwife was lovely, I couldn’t have asked for a better one in that situation, to deliver [Baby’s name], she was really nice, and she explained the options and what would be the outcome and why what was good for picking either option and what was maybe the bad points.”

STB13: “The midwife who delivered her did say to me, this wouldn’t have happened if you were a lawyer’s wife. She left. She handed in her notice. She went.”

STB23:” ‘We’re sorry about the photographs’….They didn’t realise, to a parent who’s lost a child, those photographs are everything. If we had not taken our own – actually what you’ve taken away from us is everything.. So I think that they should just take photographs of the child whether or not the parents want it, and they should try to capture every single part of the baby because you will want to see their hands, their feet, their legs. Look at their nose, their ears when the memories fade you know?”

STB05: “I didn’t have dialysis on my second pregnancy, I refused it, and my second pregnancy is still alive 13-years later.”

STB18: “The registrar I saw there then started explaining to me about how you manage urinary tract infections in pregnancy and getting it wrong! And I was just like: ‘That’s not actually right!’…I was like: ’That’s not what you’re meant to be doing; that’s what the, what the guidance is for primary care’. So, it’s kind of like you – I don’t know – I don’t want to feel in my life every time I go and see a doctor that I need to check that the doctor’s doing the right thing! But it has made me feel a bit like that.”

STB05: “The midwives that looked after us up there were brilliant. I thought they were very good, compassionate.”

STB03: “We also met with the community midwife from the area that came to visit us a few days after as well. So, that was quite good because I knew them and they came to visit.”

STB19: “My biggest regret is my Mum took all the photos of [Baby’s name], me with [Baby’s name], [Husbands name] with [Baby’s name], my Dad with [Baby’s name], there's not one photo of my Mum with [Baby’s name]. We've not got one proper family photo of all of us together so it's about giving him tips about how to make the memories.”

STB22: “That parents that have just been told that their baby has died and they’re going to have to give birth, quite simply but plainly and brutally given information such as you can do this, this is what you are allowed to do, and assumptions not being made that they’ll know that they can ask to bath her or they can dress her, if appropriate, and they can take photos and they can get somebody to come in to take photos, if that’s something that they want. I know you guys obviously do the hand and footprints, but if they want to make casts of hands etc, that almost anything goes within the parameters of what you’re allowed to do within a hospital setting obviously.”

STB28: “It was just having a bit of space and nobody told us what to do, they just gave us options and they gave us time to make decisions and we really surprised ourselves, because we did completely the opposite, we went from well my husband wasn’t sure he wanted to meet [Stillborn twin], I definitely was sure I wanted to do that so I kind of knew he would get on board with that, which he did and was grateful that he did.”

STB18: “I sort of felt like I couldn’t trust the world, like I couldn’t trust what people would say.”

STB09: “We would definitely have gone. The only reason I stayed at the hospital we did, was because of the care that we received from the consultants, so she restored that [Trust].

STB14: “I’ve actually asked to be referred to a different hospital now, because I’ve lost faith in this hospital, and the fact that I’m asking for a review and things like that. I think I’d rather just go elsewhere and, as soon as I asked to be referred to another hospital.”

STB18: “Like even when I was in labour and we knew she was dead, there’s still this bit of you is sort of excited to meet them, ‘cause they’ve been inside you for so long and you’ve kind of got that bond with them”.

STB15: “You know it is a devastating thing I think and it's having that recognition that when you're carrying a baby you have a relationship with that baby and that that's what was seemingly missing.”

STB09: “So, yeah, but then when I come home, I come home to my house and it was as if she didn’t exist.”

STB09: “I think for the longevity of care, continuity is probably one of the biggest, biggest things and also just making people feel like it was important and that it is not buried under the carpet.”

STB25: “Just after we were told, I remember saying to [Father’s name], ‘We can’t be the parents of the dead child, and that’s who we are.’ So there was obviously some sense initially of, we’re not going to survive this unless there’s some hope. I knew I was ready to be a parent, we were all set up, and then your arms are empty…”

STB26: “It took a long time to make me realise that at the end of the day I was still a mum, even if I just had one, so this was a bit hard as well.”

STB21: “As I say on 8th, everything arrived, the new baby cot, the buggy and the box from the government. We need to hide all of this. [inaudible 01:44:06] so we have got two bedrooms, and everything was there, so it was quite scary to go to. The buggy was already in bags. We contacted the shops where we bought stuff and they took everything straight away. They were super kind, everywhere, even clothes. I went to different shops saying I need to return these that I bought maybe five or six months ago. I don’t have the receipt, but I am not going to need it anymore. If you take it, good, if not I will leave it to a charity. I don’t care. It cost £2. It took me more to come here than to get the money, but I just don’t want it.”

STB22 “So given the gestation, her nursery was all ready for her coming home. I remember not being able to go into that room, the door had to be shut the whole time. I didn’t want anybody touching it, people had offered to take things out of there and we had the buggy all built in there and everything, and I didn’t want anybody touching anything, but I also couldn’t look in there either, so the door had to stay shut the whole time.”

STB25: “Beside when mum came over and before [Baby’s name] was born, but after she died and helped us pack the stuff up and my dad did, because I wanted it all gone.”

STB26: “Yeah, it’s not very nice. I think I have tried to forget about it. I was concentrating on [Twin 2] and worrying for him and not thinking of [Twin 1]’”

STB15: “. I do know now what happened to him. I wish he’d been named. I wish I’d had the opportunity to name him and because he's named to me and whether I need to do any more I don’t know.

STB25: “Yeah, and remembered, and hold their own place in your family. All those clichés that you read on the charity pages, like ‘Say my baby’s name’ is because that’s what you want. It makes your heart sing when anyone references them.”

STB19: “I said to [Husbands name] 'I can't wait knowing that she's just going to be in a fridge throughout, you know, all of Christmas.”

STB05: “When people say it, ‘Don’t worry, you can have another one’, in the days and weeks after, you’re thinking, ‘I know, but I want that one, I’d do anything to have that one. I shouldn’t be burying… my kids should be burying me’.”

**Outcomes related to investigations, knowledge and understanding the cause of death**

STB19: “So they found that I had antiphospholipid syndrome so there was a huge blood clot in the cord, and they think I had pregnancy induced lupus but obviously because I'd had a repeat test, she said it can give a false positive, a false negative so and then because I'd fallen pregnant afterwards as well, she's like, well we can't really test you for it now, but yes, so that was the reason.”

STB22: “They said she was slightly on the small side for her gestation but nothing too dramatic. Other than that, they found a few clots on the placenta, so for the follow two pregnancies I was put on aspirin and a higher dose of folic acid, but other than that, no, everything was completely fine.”

STB23: “Yeah, we went back in September to see the consultant when the post-mortem results were back and actually it transpired there was a – what was the word? – a blockage in the placenta, a rather large blockage in the placenta and that’s what caused the growth restriction, that’s what caused the blood flow to be restricted.”

STB28: “They offered psychological aftercare, but it's not something that I was really ready for then and after I left, I had all sorts of other questions, all the sorts of blaming myself questions racing around my head and I was really struggling with that enormous weight, burden of the guilt and I said I have just gotta email my consultant and just say, would this list of things have made a difference.”

STB22: “I was going to say a bit gutted because it was just one of those things and I hate it being just one of those things, but actually relieved primarily, because it meant that there was no genetic reason why we couldn’t try again and why it would necessarily happen again. So I guess yeah, more than anything I was relieved but still a bit confused of how it could still happen for no reason. But yeah, it did mean that we could try again and so we did, very quickly, which in hindsight probably wasn’t the best idea. By the time we had one of my, I don’t know whether it was the first appointment with the consultant, which would have been the post-mortem review, but bearing in mind that she was born on 1st February and by mid to late April I was pregnant again.”

STB25: “I had all these theories. I feel it was gestational diabetes or related to like virus. Like, was it chorioamnionitis or something like that? It’s gonna be one of those, it’s gonna be an infection. And the only, the only thing that was found was, and apparently it was of no clinical significance, was that there was one blood clot in one of the parts of the veins, which we knew, so in the umbilical cord. But it said in the post-mortem of no clinical significance. So [Baby’s name] died and was born in the November, and we didn’t find this out until the February. So all those months researching, contacting people, obsessing about it. And then no explanation.”

STB26: “I would have been fine with even an answer like “I am not too sure, I will have a think about it”, stuff like that, but she just answered, “You know what, it was an accident like a car crash and there’s no way of explaining that.” I don’t agree with that.”

STB27: “Because you haven’t seen it, you want to see that he was a baby, and he didn’t have any chromosome problems, because in the end after what happened with [Twin 2] they sent everything off for tests, and there was no reason. He didn’t have downs syndrome or anything.”

STB21: “That is why I am trying to go back. So many times I tried to do that, but I can’t go that far back. I try to think what I did in the morning, what I did in the afternoon. What did happen that day to see if for any reason she was trying to send me some signals, but I cannot remember anything.”

STB27: “Very unlucky, and I don’t think the care that we received from NICU, from a certain nurse who was dealing with him on that day was particularly good, not that we think now that it could have saved … if you’d have caught it first thing in the morning and stopped giving him the milk, the first thing they do is stop all feeds, there was the first instances of them being sick that night, if they’d have stopped the milk then perhaps he might have had a chance. It wouldn’t have stopped the NEC itself, because they still don’t know what causes that, there’s still no answer with that”

STB26: “Yeah, this was not – this was a disappointment for us unfortunately. Normally I think the results have to be provided to the parents ten weeks after, but we had to call the consultant maybe ten times. You did that ten times. You had to call just to get an appointment.”

STB07: “No, so there was nothing like that, that had happened. We didn’t feel like there was any reason for us to sort of file any complaints or anything like that. It felt like it was her heart and obviously having the autopsy results confirm that they can only assume that it was her heart condition, everything else about her was absolutely fine and normal and there were no chromosome defects either.”

STB19: “I think being a nurse I know too much so, you know, I know what a post-mortem entails and I just said, it's not going to change her outcome, I know it might change things for the future but right there and then I knew it was right for us, you know, not to go through that.”

STB20: “I did arrange another consultant to read the post-mortem results to begin with, because I had questions I wanted to ask. I said why at [Hospital name] have they told me it could be a placenta problem and they have given me Aspirin during my pregnancy with [Baby number 2’s name].”

STB02:” Talked to a lady who does post-mortems, not only on the baby but on the placenta as well so we discussed this extra lobe and she said it was probably a contributing factor. It was an issue, but it was, it helped the placenta become unattached too soon and it sort of suffocated the baby and it was the first time we’d sort of had any definitive sort of explanations didn’t we… A closure. Yes, an explanation because there’s so many babies that are still born that are unexplained (coughing), you know I mean just no-one ever knows what happened so at least we sort of had an almost a reason for what happened.”

STB02: “Yes. We were told placenta abruption, one of those things, 10 percent change of it happening again which to me was a 90 percent chance of it not happening again which I quite liked the odds. On the next pregnancy we would be monitored very carefully, more scans which to be honest I thought well we’d already had all the scans anyway.”

STB04: “She just sort of went, “Well, yes obviously she was Downs”. “What? We didn’t know that”. And it was just like having a … it was like a second loss because I’d just … you’re trying to get your head around you know, this baby that you’ve just loss and now there’s a whole new thing. I know I hadn’t kind of chosen to have the test, but I didn’t think … you know, when you saw her, you couldn’t see. She looked like she was a lovely healthy baby. Then they said she had holes in her heart which probably could have been as a result of the Downs and then I remember … I was just a bit gobsmacked. She was really cold, and it was really matter of fact. There was no kindness, then I remember asking was that why she died. “Oh, well, no, probably not. We don’t know why she died”. It was just sort of like; you’ve literally delivered this huge bit of news to me. You’ve still left me with we don’t know why. Then I remember thinking is there not a link? Surely, is there not a link? Then we were kind of just like shuffled out.”

STB26: “This was a disappointment for us unfortunately. Normally I think the results have to be provided to the parents ten weeks after, but we had to call the consultant maybe ten times. You did that ten times. You had to call just to get an appointment.”

STB06: “The only thing the post-mortem showed was placenta deficiency, which the consultant suggested was a way of just saying that they just don’t really know, which I guess in some ways was a relief because it meant – well, I don’t know. It made it quite hard because it wasn’t like we found a reason or something that we could check for in subsequent pregnancies.”

STB07: “She had a heart condition and then maybe there would be something in there that could be used and pulled into data and maybe into research later on rather than just not being a part of it. Hence why us doing this as well and speaking about our experience and hope better outcomes for other people to the same with the autopsy.”

STB08: “They said that she had died 24 to 48 hours before I’d given birth, though most likely 24 hours before which meant sometime in the early hours of Friday morning. So on the Tuesday when she was experiencing distress as they now know, she was still alive. They said that mistakes have been made and the person that had taken the call had been identified and did I want to take the matter further. I spoke. ‘The only thing I want is better training,’ so that they don’t put another woman through what I went through but to be honest if the midwife knows that her saying to me, ‘Well we don’t want you in here when you’re throwing up,’ had led to the death of my baby then I very much doubt that she’s going need anything else and I didn’t want any compensation because nothing’s going to bring her back.”

STB10: “Well they didn't find a reason why she died, but I'm glad that we had the post-mortem, because we wouldn't have been any the wiser otherwise. I think if we hadn't gone ahead with it, I would have always thought, oh we might have found a reason if we had. So I definitely think it was the right thing to do.”

STB11: “They said that she died through – she had three blood clots in her cord and obviously that killed her. They were so certain that would have been it, because I have seven healthy boys, they said that it would have been so rare to find a condition in [Baby’s name] that it wasn’t really worth bothering”

STB14: “Yes, we did, yeah. Because we wanted to have answers, but also, I thought it might help somebody else, because they said they do a lot of, they keep slides for research and stuff. So we thought if maybe we could stop it happening again to someone else, that it would be worth something would come out of it. Yeah, and I’ve been back for another appointment to ask more questions because I think sometimes you can’t think of all the questions at once, and they’ve been good about answering them.”

STB15: “And when I got the certificate, the stillbirth certificate, it said intrauterine death, placenta insufficient, placenta insufficiency, which did make me cross really because when I went on to have my daughter in fact, I remembered the bit about the placenta was a bit small, but I'd never seen the insufficiency bit.”

STB15: “I suppose it's like anything else, it's easier if you understand why and I know sometimes there are no answers but if you get someone understanding why you need to know why, you start you know I can remember thinking what did I do wrong, it must be my fault, what did I do during the pregnancy that caused this and I remember there being something in the paper some time afterwards about a higher incidence of stillbirth if you lived near an airport. This would have been in the 70’s I think. Well of course I did live near a major airport, but then lots of babies were born where people lived near the airport, so I don't know whether that was ruled out or not.”

STB17: “For other pregnancies I suppose, but no, not really because it just made me feel more devastated that he was perfect. Why did that happen? When we got the paperwork back it just said unexplained, so I suppose in my mind I think it was the cord because that was mentioned at some point, but who knows?”

STB18: “Well nothing really more than what we already knew, which was there was an ascending bacterial infection. They did find evidence of that in the placenta and on [Baby’s name]’s skin, but they don’t know for sure which way round things happened; whether it was a bacterial infection in the placenta which you know, caused the problems, but they think probably most likely was that happened afterwards, so my waters broke first because of the bacterial infection I had and then that... because there was no longer that protection, that kind of infected the placenta and then [Baby’s name].... so there’s still some sort of unanswered questions around exactly what happened.”

STB01: “I feel if we ever get pregnant again I will have to take all of this into my own hands and will have done research myself and I feel I wouldn’t get the support that I would need in this hospital. I will have to do it through the NHS because we can’t afford to go through a pregnancy privately, but I’d have to research a lot and maybe take on somebody that’s can consult with us during the pregnancy as and when needed.”

STB25: “Whereas all the other stuff, and there probably was an element of distrust in our own hospital, because [Baby’s name] died on their watch, so yeah they were there when it happened, but I was frustrated that I had been told silly things, like drink orange juice and babies don’t move as much. There was a lack of appropriate information that now is a given.”

STB21: “First of all, care for parents has to be before this happens. The idea is to try and prevent these things. You won’t have a job!”

STB03: “We are aware about miscarriages because it’s more common in the first 12 weeks, everybody knows about that, but nobody talks about things can happen after the first trimester …compare it to when we go for an operation, people always say, “Ok, this operation has some risks”, but we don’t realise that a pregnancy is a very random thing, and anything can happen from a pregnancy.”

ST01: “I didn’t really get any answers. She was quite abrupt with discussing things with us. She was quite dismissive in the sense I was asking questions because I don’t really understand all this jargon and I felt a bit silly like I was asking silly questions.”

STB13: “She said there was a true knot and I said what do you mean? And she said she had a true knot. It’s in her notes and I feel very angry that that wasn’t shared with me. “

STB28: “Everything was perfectly normal and that’s even worse because you are left with blaming yourself …you need an answer and you can’t just accept it just happens, it's so hard to accept that… You are just constantly searching for an explanation and the only explanation could be it's me, because I am the only one, I am responsible.”

STB01: “So we ended up taking the same notes and going to a private doctor to analyse and literally sit and have a conversation with us and that was so much better because she sat and went through it all in layman’s terms exactly what had happened.”

Interviewer: Did you have any formalised review on your case?

STB06: “No, and I think that was something that upset me. The consultant was very nice, and we had the post-mortem, but I guess at that time I just wanted more answers and I felt it was a bit odd that stillbirth is very common, but still nobody really knows about it, and why wasn’t my data being plugged into some kind of study where it could actually be used to find out answers? Or even, like you say, in a case review, which as far as I know didn’t happen or didn’t happen with any input. Nobody was asking me about my pregnancy. They asked all the kind of normal questions, ‘Was there anything abnormal in your pregnancy?’ but nobody asked me in any more depth about anything.”

STB09: “When I got this Root Cause Analysis through basically saying all the areas that had gone wrong and I wanted to speak to somebody. I was told that I could go through a timeline of what happened, because they are saying, that is what happened without any input from us. They could effectively put what they want down… I have got a letter saying that we will go through it and we didn’t go through the Root Cause Analysis with anybody, nobody.”

**Outcomes when a stillbirth occurs in a multiple pregnancy**

STB27: “Not at the time, because it was all too up in the air, because they weren’t sure how it was going to progress. Whether the people at the specialist hospital knew he was going to die so they referred me back to our own hospital thinking, “It will get dealt with as the pregnancy progresses.”

STB26: “Yeah, through this the consultant told us it would require close monitoring because it’s high-risk pregnancy, so we had fortnightly scans. They were growing okay, and they didn’t have the baby transfusion syndrome. This was going fine, so up to 32 weeks, and so we had a scan at 32 weeks.”

STB26: “Yeah, the next day the consultant came back, she had discussed with the fetal medicine unit and they advised to wait so she said she was – actually because [Twin 1] probably died five days ago then [Twin 2] is probably not going to die, so there is low risk this would happen, and it’s higher risk for – basically being premature is higher risk than a delivery – him dying. She said better to keep him for now and they’ll do a C-section, she said by 37 weeks.”

STB27: “Because even though they were separate placentas and separate amniotic sacks there was still a risk to [Twin 2] which I think they downplayed to us, did they? Because it was only after [Twin 1] died and they did all these checks on [Twin 2] and they were so pleased he was fine we realised actually that was quite a big risk for him.”

STB27: “So they did all that, that was fine, [Twin 1] was born, they carried him away and then they got [Twin 2] out and he cried. RES2: He cried, and honestly, we were so relieved.”

STB28: “because I had a few crushing moments when I just thought they were acknowledging that he was a twin and I was really grateful, but they weren’t actually so the fact that it still says twin on his red book I am pleased about because I don’t want that to be forgotten.”

STB26: “Yeah, this was something as well, so we had a lot of support for us after the birth in terms of bereavement and stuff, but nobody really advised us what we should say to [Twin 2] or what we – how and when’s the proper time, so we’ll have to see how we’re going to. We’re still not sure but we’ve got memory box with everything for him, like the hand and footprints. We’ve asked as well for all the scans, so we’ve got a USB stick with everything on it.”

STB26: “[The consultant said] We could do an MRI scan the following week, it’s going to tell us how he is, but it is not going to predict if is going to be alright, physically all right, but just confirm if there is something big so we could see…. Like if he was badly brain damaged what we were going to do…He did explain there was 25 percent chance of this happening so it’s quite high risk.”

STB26: “Mother: She also said that now we were back to the singleton pregnancy which –

Father: …was quite inappropriate.

Mother: We didn’t say anything to that. It’s still twin pregnancy. [Twin 1] was still there and I would still have to deliver him, and he was impacting [Twin 2] as well.”

STB26: “I couldn’t say, again I didn’t react, that was really weird because I was really relieved that [Twin 2] was there, we managed to make it to thirty-six weeks. This stuff was finished. The pregnancy was finished, and we can start our life again, but at the same time at that point I knew [Twin 1] was gone forever.”

STB26: “Maybe that’s weird for us because we were expecting two babies and we still have one so I think we are fully concentrating on [Twin 2] so maybe, hopefully not, maybe it will come later, and we will fully realise.”

STB24: “I went straight to my mother and asked her and her first response, I guess she was triggered was just to shout at me and send me away. That was sort of quite reinforcing that I’d done something wrong and it was my fault, at that age. It was a subject that was never allowed to be mentioned.”

STB26: “Like how to explain to him that he lost his twin. Maybe I would have liked, I don’t know, to get to know, probably would never know, but what could be the impact on him, not in terms of matter of fact, but in terms of psychological effect because he lost hist twin, if it’s going to have any impact on him, I don’t know.”

**Grief and psychological outcomes**

**Grief outcomes**

STB19: “I was completely shocked I think, and I didn't really know how to react because it was almost like, was she joking?”

STB09: “your world just completely stands still, everything stands still, and you don’t know where you go forward. You’ve got no hope, everything is on the edge. You don’t really know where you are?”

STB15: “And I can understand you know because they have, they've lost their future.”

STB02: “I think it was that typical guy thing about coping too much and not letting yourself grieve or you know be emotional in front of anyone and I think it’s a harmful thing not to do that so if I ever give a man advice, I know he might not take it because been a guy, it would be to grieve as much as you can when you can otherwise it affects you physically.”

STB08: “Yeah, I think it was the summer so when Tilly was tiny, I cried a lot. I just, was so tired and it just felt really hard, really… I was so glad we had her when we did because if I hadn’t have fallen pregnant quickly, I would’ve got too scared to do it because it was terrifying and one of the reasons, we decided no more children.”

STB20: “I wasn’t actually processing the emotions I felt about it, I wasn’t really taking time to actually sit down and think about what happened and think about her and I then I feel like when I got pregnant it all just hit me and I felt I needed to get stuff out.”

STB08: “I literally didn’t understand what he was saying to me. He said, ‘There’s no heartbeat to be found because there’s no heartbeat there,’ and the words just didn’t make sense to me and I just suddenly thought oh no I think my baby’s died and I felt so guilty because if I’d have just gone in three days before she would have been alive.”

STB09: “Looking back now with [Baby’s name], if maybe I’d have asked more questions, maybe a different course of action would have happened, and it wouldn’t have led to our outcome? That’s always going to be the things you ask yourself down the line.”

STB09: “I didn’t want to see anybody for such a long time, because I was almost, I know this sounds horrible, but I was almost embarrassed that I couldn’t do the one thing that I am designed to do. Not designed but it was my job was to get my baby out safely, that’s what I felt. It didn’t matter what anybody else said to me, loads of people I'm sure will have a view or have something to say in response to that but that was regardless. My job was to get [Baby’s name] here and to get her here safely and I couldn’t even manage that. For me I just felt like a complete failure.”

STB09: “Because of the way that it happened as well, I felt like I’d suffocated her to death. She was under the stress and I felt like my body suffocated her and that has been… then you're looking at not only losing the most precious thing in your life, you're actually then feel like you’ve caused it.”

STB10: “I suppose that was a lot of my guilt as well was that I should have been able to look after my baby and why didn't I... why couldn't I look after my baby when I look after so many other people's babies and children.”

STB13: “I’ve suffered from depression ever since, anxiety as well and there are times, I think I’m not – yes it would happen to me because I’ve done something where I’m not a very good person. You know all these stupid thoughts, but it does come to you. I deserved it.

STB13: “Been frightened to go there in case it’s something about me cause I’ve looked for every way to blame myself, but I mean I’d lost incredible amount of weight, I’d given up smoking, I hadn’t drunk, I hadn’t even taken paracetamol. Nothing. I did nothing that would jeopardise my baby. Absolutely nothing. I can remember [Ex partner’s name] saying after she was born, he went outside for a breath of fresh air and there were young girls heavily pregnant smoking in the doorway. Said he had to walk away

STB14: “I’ve tried to think of loads of things, I’ve researched, is it that I ate too much sugar, because they talk about pre-diabetes, the doctors can’t tell me so I’ve sort of blamed everything, maybe my inhalers, anything that I can think of. But we don’t know.”

STB17: “I was isolated and then I felt so guilty because I was struggling with her. Because I was on my own and she was crying all the time and I was thinking, “I don’t want to be ungrateful because I would give anything to have [Baby’s name].” I remember for the year or two after him until I had [Second child] that’s all I wanted was for him to be screaming his head off and me not getting any sleep and feeling like a new mum.”

STB25: “And yet a natural reaction to bereavement is to tell yourself you were wrong about some part of it. I think however it plays out, mums and dads think that they did the wrong thing.”

“From that then becomes the journey and the story. That was the situation. That’s also busy and there's so much going on that it's everything from that point onwards that is the journey”

STB02: “I’m aiming for his 18th birthday that his name would have touched the world in some small way, whether it be SANDS now and people have written his name and just put it next to a stone so that his name touches the world before his 18th birthday so that I can do a photobook of all the places his name has touched.”

STB02: “Yes my mum was very affected by it and my dad, so my mum is a bereavement counsellor, so she had a lot of time off from doing that and she also like runs a playgroup, so she didn’t do that for a few months.”

STB15: “I think that was particularly hard for my parents when we drove home after just before New Year we'd been, I'd been living there for six or eight weeks and I can see as we drove out of their drive, I can see my mother's face crumbling because it was so different from what it was expected to be. So I think she found it pretty hard.”

**Mental health outcomes**

STB05: I did get some counselling eventually, like five years down the line. I’d got to the point where my brain had gone, that’s it. I wasn’t sleeping, I’d got insomnia, and I was getting teary all the time, I wasn’t coping. Luckily my renal consultant said, ‘[Wife], is it because you’re going as mad as a box of frogs?’ I said, ‘Well yeah, I think it might because I’m losing the perspective basically. I’m not sleeping, and I’m basically thinking am I going to go to sleep and not wake up, thinking I’m going to die anyway, I was losing the value of life anyway. My consultant said, ‘Look it’s about time we got you some help, some sort of cognitive talking therapy’.

STB07 “So yeah it was through the GP and I just started, I was sad and not clinically depressed or anything, but you know something’s definitely got to change with my mental wealth and my mental state and it was just a bit”

STB16: “I saw the GP just before my marriage broke up and, again, it was my ex-wife dragging me there, saying, “Can you fix him?” I only went because it was a condition otherwise, I’m going and I went for that reason and he gave me some antidepressants. I didn’t want to take them, but I remember we were going away to Florida and I took them for two weeks while I was there and I didn’t take them, I pretended to take them because she wanted me to, and I didn’t want to take them. I knew I didn’t want to take them, and I didn’t need them. I didn’t need them, I needed something else, and it was only then when I came back and I decided I wouldn’t take them, but I would sit in the counselling room, which was the other option given to me, I suddenly thought I needed a counsellor.”

STB28: “Probably maybe a couple of months later and because everybody kept saying you must make sure you see someone, and you must talk about this and I felt at the time like god well I might go mad if I don’t because is everyone is saying I must, but I don’t really feel like I wanna talk to anyone.”

STB23: ‘Most people find Christmas is hard. Have you thought about how you’re going to deal with it? When we were going out with our friends, I said to her: ‘We’ve got this first meeting up with all of our friends. It’s going to be a big evening out and some of them we haven’t seen since this has happened.’ And she said: ‘Have you thought about what you’re going to say and how you’re going to approach it?’ And I said: ‘No but I won’t lie, I am anxious about it because it’s going to be a difficult situation

STB 19: “I'd been referred to a counselling group that dealt with bereavement and I went to the first session and it was more about people who'd been affected by death. So it's a group setting and there was people there who had - there was a 40-year-old couple who'd lost their Mum and then their Dad had died shortly after. Somebody's son. So it wasn't specifically related to baby loss. And I just remember sitting there thinking, this isn't right, it's not the right place for me, I just felt really out of place. And we went to do like a group, I don't know whatever it was, and I just remember getting up and walking out and the lady came out after me and I just said, 'my grief is different to their grief,' and she was sort of saying, 'everyone's grief is different.'”

STB10: “It was just a bit of a chance to say things to somebody who was used to hearing the kind of things I was talking about and somebody who wouldn't judge me as well for the things I was saying. She also had quite a lot of practical suggestions for things and particularly in relation to our older daughter and how to help her as well.”

STB14: “Yeah, I had six sessions of counselling that I was offered. It was really helpful the first couple of times because it was getting me out of the house and talking to somebody.”

STB18: “I think having a fixed time in the week to express my feelings about things and what’s going on in my life, and it’s a sort of protected time when I know that I can do that and focus on grief. It’s sort of uninterrupted and I don’t have to think about anyone else’s feelings, you know, it’s just, it’s my time. I think that’s really important. It’s been really, really helpful for me anyway.”

STB23: “Yeah she’s made us realise that it wasn’t just losing the baby, it was the whole process of having to make a decision to end the baby’s life, and she made us – we never had any doubt that it was the right decision – but she just sort of helped us feel that we shouldn’t have any guilt and we were doing it for the right reasons and for [Baby’s name] really. Which helped me a lot as well because although it was the right decision, it’s still something that goes in your head … the what ifs?”

STB13: “were referred to the bereavement counsellor at the hospital and out of all of it, it was like a macabre. I’ve got a macabre sense of humour. I sat there thinking you funny little lady. She was like something from French and Saunders with this textbook, you know this sort of sympathy and I couldn’t cotton on to her at all.”

STB13: “I went for counselling. They offered me counselling and I had to wait in the antenatal clinic which was full of people I worked with because I worked in the family centre so there were women in there that knew me from my work because I was on maternity leave, very pregnant themselves – what did you have? And when I walked in and I said I think this is pretty disgusting that you’ve made me sit there and humiliate myself with people that know me professionally, knowing that I couldn’t respond to them because they’re heavily pregnant themselves.”

STB17: “Whereas a health professional maybe would know the right questions to ask, I don’t know, just make you feel like you have still had a baby, you’ve still been through a pregnancy, you’ve still experienced stuff that other people have experienced and it’s okay to talk about it.”

STB03: “We had good support from the midwife so they’re sort of counsellors because they were there supporting us but having a professional counsellor to maybe come and see us after a week or so and then see how you’re doing mentally and how things are going, would have probably been useful and then understanding the need for the counselling.”

STB18: “I think actually in this pregnancy it would have been really helpful to have some joint sessions with both [Husband’s name] and I, especially more towards the beginning because I think he didn’t really understand the impact. I think for him it was like: ‘okay you’re pregnant again, start again’ kind of thing, whereas for me it was like: ‘back being pregnant again’ and it was really difficult and I think he didn’t really understand that. So there have been times when I thought it would have been really helpful to have some joint counselling sessions”

STB27: “Somebody who maybe checks in with you, because I’m not really one for pushing it, and a lot of people wouldn’t be, so it would be nice if she would maybe, even by email every six months or even on their birthdays, “I’m just checking in, I noticed that it’s his birthday, how are you doing this day?” Sometimes, you can’t have counselling right after the event is what she’s said to us, because almost you need to process it, it’s more at this sort of stage there may be benefit from it.”

**Emotional outcomes**

STB09: “I've never felt like I've had so little control over myself and my emotions.”

STB18: “You almost feel like it’s vanity or that you shouldn’t be thinking about [weight gain] when you’re pregnant or when you’ve just lost a baby, but actually your body and your relationship with your body is so important and it’s an important part of your identity.”

**Whole person outcomes**

STB02: “We gotta be resigned to the fact though that they’ll always be something sort of wrong in our lives from the amount of time we’ve been with people who’ve lost babies years and years before us.”

STB09: “: We were trying to get back to a normal life, weren't we? RES2: We thought we’d try and be normal, but obviously we realised that there was no normal.”

STB20: “I feel it's really changed me; I don’t remember the person I was before having [Baby’s name], I don’t remember the last time I just felt like I had no worries in the world.”

STB25; “It made me reflect on how I behaved around bereavement. I hadn’t been great actually, so as time went on, I wasn’t as harsh in my mind to people about it as I was initially, you should give people a chance.”

STB26: “It helped me to understand more how pregnancy and to have children is not an easy process, although it could seem to be – especially to men, it’s helped me understand how fragile it is always, so maybe a bit more sensitive to pregnant women’s concerns, also because it was the first pregnancy, so it all happened at the same time.”

STB08: “Yeah, life is different, it’s a new normal, and it is not the same it’s a new way of living.”

STB09: “When something like that happens, it becomes the new normal and sometimes it is quite hard to break what is normal. It became normal for us not to go out and not to socialise and to feel sad and to be around me that was sad. He kind of got into that as being his new normal.”

STB26: “I think it changed me, definitely. I used to be – I’m not really good with feelings usually and sometimes I didn’t really understand why people get upset with stuff, but I think I’m better with that now, so I guess it’s positive impact. I went through something that’s dreadful, it just – now I understand people a bit better.”

STB23: Res 1: And it also makes you feel a little bit – what’s the word?...that nothing can harm you anymore.

Res 2: Untouchable…like we can get through anything.”

**Social and family outcomes**

**Social outcomes**

STB10: “But from his perspective he's talked to me about this, the horrors, that he was left holding her and there was nowhere in the room for him to put her, there was no cot in the room. He wasn't able to call a bell or anything.”

STB14: “I think they were doing the best they could, given the staffing and conditions. They were very sensitive midwives when they came in, there was a lot of rotation of staff and obviously there’s women in labour who are going to have children to take home, to me I felt I was a lesser priority, so we were left a lot of the time.”

STB15 “The birth happened towards the end of the afternoon about four o'clock and I can remember the birth, but I didn't see the baby. Then they just left me alone and I think that was the hardest thing that nobody talked to me.”

STB15 “Nothing, nothing was offered and all I remember when I was in hospital was being on my own and it was you know a devastating experience.”

STB19: “I felt very isolated being in this side room near the entrance door, it was almost like we were an afterthought.”

“I tried not to buzz because I didn't want to disturb the ward because I knew they were busy.”

STB20: “I just remember lying with her [inaudible 10:57] like a really long time and my ex-partner had held her for a while after, but he left the hospital that night, didn’t want to stay with her [inaudible 11:13] I was just scared [inaudible 11:19] it just felt really scary.”

STB23: “And what made it even harder was... after that you’re just left on your own not knowing what’s happening afterwards for just absolutely hours, not knowing – we were sort of expecting someone to come through like – ‘this is what needs to happen now’”.

STB23 “To communicate. Just like there was a time in that hospital where we were probably left on our own for a good five hours and this was after the baby... fair enough if the baby’s with us and we’re holding the baby and having a bit of time with the baby, but after the baby’s gone we’re just sort of sitting there.

STB23: “What do we do, what do we do now? Where is she? What happens now? It was, what happens now? Do I need to do anything?”

STB23: “When that woman came in and said: ‘I’ll call you and we’ll go for a coffee’, I thought: ‘yeah okay that will be nice, that will be nice, that will be nice’, even though she wasn’t there, yeah that would be nice. When she didn’t, I got angry and frustrated that actually the offer at the time, yeah that would be good. That is good because I will be knocking around at home not at work. When you’re knocking at home suddenly and nobody’s looking after you or looking out for you then yeah, you feel really let down and very lonely, very lonely.”

STB10 “Then because we went away for [Baby’s name]'s funeral, we then didn't hear from the bereavement midwife at all and I think... I think I would have expected her to be in contact more than she was. So I felt slightly let down with that aspect of care… I think I was just a bit too vulnerable to try and chase up on it at that point. Then it was another couple of months later when I started to feel, no I really need to look at these notes, or have somebody go through them with me. So I texted and then I emailed her eventually and eventually managed to get an appointment to see her and go through the notes.”

STB13 “I think the doctor could have come and seen me. I wonder who that anonymous doctor was. Never told. I asked and I wasn’t told.”

STB10 No, I didn't have any more contact with her. I think I probably could have done with much more contact from her than from an obstetrician. I don't know quite what I expected but I think I maybe expected her to come out and see us at home more or to... it would be nice to have a phone call to check how we were doing and offer some further support or counselling or something.

STB15 I think the thing that I feel so cross about is that there was absolutely nothing, no care whatsoever, absolutely no, nobody came to see me, and the nurses didn’t talk to me. I’m not saying it was particularly unkind, they weren’t, but there was no kindness there

STB17: Yes, I did feel like there was no – it was literally like that happened and then there was no support from any health professionals at all, we were just left struggling really.

STB19 “I don't know. I really don't know. I was very - I think because I'd been sent home and pretty much left to my own devices, I then developed this very, you know, I can deal with this by myself actually. Basically, if they don't give a shit about me why should I give a shit.”

STB10 “When my GP said to me, 'come in for a welfare visit,' it's not something that I would normally do, I was more than happy for her to make the appointment and I just turned up on the day. So I think had it been dealt with you know, I don't know, within the first eight weeks, maybe. If something had been sent out maybe within say, 20 weeks maybe but then I think anything after that I think you just have a 'stick it up your arse' attitude. Which is probably, you know, not helping me, sort of almost like you cut your nose off to spite your face, and I think that's very much how I became. It's a bit self-destruction I know, but I had nothing.”

STB23: “Maybe contacting some form of support on behalf of the parents, so they contact the parents themselves rather than the parents, with everything they have to deal with having to try and find their own support – waiting for leaving voice mails, waiting for people to call back who never do, because it just become demoralising.”

STB01: **“**I could talk about what had happened which strangely enough I really wanted to do a lot of. I wanted to tell them exactly what had happened, and I wanted them to know.”

STB06: “I don’t know. I think it is important to talk about things.”

STB07: “It’s really nice to talk it out, talk about – even a couple of years down the line now and it’s nice to talk about her as a positive rather than just a negative.”

STB08: “I think it’s important that people talk about it and I often have people say to me, ‘Oh you’re so brave’ and you’re like, ‘I’m not brave. I’m just doing what anyone would do. I’m just getting on with things,’ but they say, ‘Oh you’re so brave talking about it,’ but it feels wrong not to. She was my daughter. I will talk about her until the day I die because she’s important.”

STB10: “I love it when people speak about her. But sometimes it was a bit of an elephant in the room and I think I've had to get better at talking about her myself in order that other people are less worried about talking about her I guess.”

STB12: “My advice to people who are supporting somebody would definitely be to like talk, you know like don’t shy away from it, like just sit with them and be like – yes it’s really sad and it’s really awful and there’s nothing you can say that will make them feel better but just having somebody to sit there and listen, just tell me how awful it is.”

STB14 “So it’s like you’re excluded from those conversations, whereas other people have been through it and they go, oh how long was your labour? Who does she look like? Things like that. I almost wanted to be asked something because she was our daughter and I wanted to share what we did have with other people.”

STB15: “I would know now if that happened to somebody I knew or even you know a child death, not necessarily a stillbirth you know how important it is to talk about it.”

STB15: “My advice would be I think to talk about it, not to brush it aside and not to accept just oh go off and have another one, not to just leave, just draw a line under it.”

STB17: “All I wanted to talk about was [Baby’s name], and no one wanted to talk to me about [Baby’s name] I suppose, so just to go through the experience, because even like the birth and that, I felt like I never spoke about it again really unless I found someone that actually wanted to talk about it.”

STB18: “I didn’t want to kind of feel like I was hiding away and like not talking about it when it was such an important event.”

STB20: “I had to just start doing that because I just refused to let anyone make me feel like she was gonna be forgotten, that was always something that was really frightening to me and something that I would never want us to just pretend, I couldn’t imagine my life just pretending that she wasn’t here. I have got a little teddy bear that is her basically, so we cuddle with that every night and I have all her pictures.”

STB23: “The only way actually you’re going to help people is by talking about it because that’s what makes other people talk about it. If you talk about it you encourage your friends to talk about her or you know, you encourage your friends talk about your child, you encourage your family; but we shouldn’t have to educate people to talk about it just because it was our loss, you know like we shouldn’t have to be the ones who talk so freely about it just to make other people be able to talk freely about it, if that makes any sense.”

STB24: “One of the things I found, especially with the men, the old men in the Lone Twin network. They don’t have anybody to talk to. They don’t talk to it about anybody. That’s the thing, they don’t talk because who wants to hear it? So, talking about it, is so, so important.”

STB25: “It’s actually really lovely to be able to talk about it, because no one ever wants to hear the birth story, when you’ve had something like this happen.”

STB27: “It is and it’s what we live with on a daily basis, it’s not a secret, and we’re very open with people when we talk to them.”

STB01: “Knowing that there are people out there who can help you with a little bit of counselling and talk about things and things you can’t talk to your friends about then you know just do it you’ve got nothing to lose.”

STB01: “I think it would have been good to have someone either to I know that they can’t have someone to come round but if they’d said just pop over tomorrow morning and the bereavement midwife, we’ve got here already she’s going to talk to you about what’s going to happen.”

STB03:” Even though there was not much to discuss with them because it was a bit strange to talk about myself to be honest, about my own health, because I was just not thinking about it at all. It was good to have them to sort of remind me how are you feeling? How are things going? And having the opportunity to talk about things.”

STB18: “Well is there anyone I can talk to at all?’ and she said: ‘Oh well I can, you know the chaplain, you know you could talk to the chaplain’ and I think I did speak to the chaplain once on the phone, but that wasn’t what I really wanted. Like what I really wanted was to have like a face-to-face chat with somebody and they just couldn’t offer me that and so that was quite difficult because I felt like once we left hospital, we were just kind of left on our own and I didn’t get any six-week check. I got one midwife who phoned me the day after we got home and she basically sounded like she didn’t really know what she should do or say and she just sort of said: ‘do you want me to come round?’ and I said: ‘no.’ I was like: ‘my mum’s here, my husband’s here, there’s nothing... no’ And she was like: ‘oh well you know where we are if you need us’ and I was like: ‘okay’ and then I just never heard from them again.”

STB15: “Then they just left me alone and I think that was the hardest thing that nobody talked to me.”

STB23: “Everyone should have access to somebody. Everyone should have access to a midwife that has seen this. Because that’s what she is. She’s a midwife that has seen it happen repeatedly and isn’t frightened to talk about it; and has seen how families feel. Isn’t frightened to ask or to say: ‘how do you feel? And How did it make you feel? Did you feel this? ‘And encourage us to talk because.”

STB03: “I came on reduced hours initially as well, with the support of work, so that helped then I felt little by little phased back to full time work, which was quite good, very well managed. I would really recommend that for any work situation which I know is not maybe very common in every company, so you just went back to normal.”

STB04: “So I went back to work after six months. I think I was just existing for a bit really to be honest. I wasn’t really, but yeah. I probably wasn’t ready at that point really.”

“, I probably was just in this kind of fog for probably quite a few years in retrospect. I was in a fog; I was just existing really. You know, you lose your way a bit.”

“I do remember kind of being made aware that I was entitled to full maternity because of the age, because of the point of which the baby was lost. As I said, I didn’t take the full … I don’t think it was a year by then. I didn’t take the full amount. I went back after six months. I went back in the September.”

STB05: “you’re constantly having to go over, ‘What did you have?’ ‘What did you call your baby?’ Having to go over and over the fact that your baby died, it’s really hard, really hard. It came to a point where I had to speak to senior management to say, ‘Can you just send them all an email and tell the whole centre, this is what’s happened, and that I’m happy if they want to talk to me, ask me stuff, but I just don’t want to be answering every day to numerous people, because I find it really difficult’.”

STB06: “I guess when you’re pregnant you’re maybe not performing at your best, and I feel like I’ve had an extra year or not performing at my best. I think we would probably have only had three children anyway, but I’ve had four lots of nine months of not really feeling particularly well, which I guess when you have those all quite close together maybe you do feel like you haven’t done your best work over that five or six-year period. But I think, in the end everything’s kind of worked out okay.:”

STB08: **“**He hadn’t done enough time there to earn any time off and he was treated as if he had a zero hour’s contract, so not entitled to sick leave or any sort of leave. I needed him to take two weeks off.”

STB09: “I was working a lot from home, it was good in the essence that it took my mind off it, but I was too anxious to be around people and it enabled me to hide for longer because I could stay at home and work.”

STB10: “I also found going back to work really, really difficult. It was probably the most challenging thing that I did. So having their support there for when I went back to work was brilliant.”

“STB10: Just things about trying to have a phased start and being supernumerary and trying not to have too many expectations on myself to begin with. Then kind of practical things which they had done when they went back and also mistakes which they had made themselves. So one girl who felt like she'd probably gone back too early in retrospect and regretted that. So I took my full maternity leave and I'm really pleased that I did.”

STB12; “No they weren’t very good at all. I got told 14 weeks after I lost him, I got pulled in for a meeting and told that my job was been made redundant and then I ended up agreeing to take a different job which was three days a week rather than five and it was a pay scale lower and then actually the jobs didn’t get made redundant and, in that meeting, I got upset and they asked me why I was crying? Was it because been in work reminded me of a happy pregnancy? I was like, no it’s because 14 weeks I didn’t think I’d be sat here talking about returning to work.”

STB14: “So up in the new year I wanted to get back to work and just be busy, so I wasn’t thinking constantly about things”

STB16: “I went back to work and people said, “You shouldn’t be back here,” but it’s exactly what I needed. It’s the one thing that I needed but, looking back, I think I probably should have taken a little bit more time. I don’t think I really thought about myself too much in those days. It seems a strange thing to say and it sounds very noble, doesn’t it, but I don’t think I did think about myself an awful lot.”

STB16: “I went back almost immediately after the funeral. I needed that normality of getting up again but also I needed people to help me with [Mother’s name] as well.”

STB19:” thinking about going back to work, so to me it was almost like normality was getting back to our kind of level of normal.”

STB21: “I think also to explain this at work and to send a message to my manager. We asked everybody not to reply. When you say, ‘don’t reply’ what does everybody do? Call, messages, but we didn’t read anything for weeks. Our mailbox was full. We knew what people were going to ask and we weren’t ready to give answers. To people who are far away, it is very difficult to keep calm. People can’t understand what is happening. Even us we didn’t know what to do. You also contacted your work.”

ST22: “So I ended up staying on sick leave, I didn’t take any maternity leave, I just had sick leave instead. Actually, that made me feel much better, I couldn’t bear the thought of being on maternity leave when I didn’t have a baby at home.”

STB23: “Oh for me absolutely, I lost, I dropped my workload completely I think, and I think I’m still catching up with it now.”

STB25: “When I went to my occ health appointment, she said, ‘Enough. You’re just being treated for PTSD; this is not the right time.’ Because of the nature of the work I did, which I wouldn’t describe, but it was just too near the mark with what had happened to me. So I stayed off, and then I was on maternity leave with [Second baby’s name], so I only went back to work in 2017, so I was actually off four years.”

STB28: “I was just on my standard maternity; I went back after six months. Work were very good, because certainly from going back to the time I went off with [Subsequent child] I would have to go out of the office and cry a lot. They were seemingly very understanding.”

**Partner and family relationship outcomes**

STB21: “I think so, yes. It definitely hasn’t put us apart. We are either at the same level, or even closer, especially talking about this. For other things, our daily things, we still have, but when it comes to this and the way we think about stuff, we think the same way. It made us a bit better.”

STB27: “I think so, and I think it’s made our relationship stronger as well, I think we were talking to people and experiences like this either make or break families and couples. I think it’s either make or break for people, but I think we’re probably now stronger than ever, we been through this situation.”

STB28: “I am still just envious of how brilliant my husband is at being emotional on cue, gotta work on that. He would cry all day, not be able to hold down a conversation and sleep like a baby at night, whereas I would be perfectly fine in the day and then wake up at four in the morning and have to kick him awake, actually now I am not feeling so strong, sort of comfort me but he would be snoring and whatever, so it's interesting to see how differently it affects u)

STB26: “I just did my best to try and reassure her or to support her to go through those weeks until giving birth.”

STB23:” I think a lot of the reason when you do stay together is because you realise that there is nobody else that knows this experience. That nobody else has been through this with you and it would be almost terrible to lose the person, the only other person who knew your daughter or your child.”

STB10: “So he... even when we were still in the delivery room he... and he was in tears, he said this is probably the only time I will cry, but don't worry about that, that's just me and just how I deal with things. We have dealt with things very differently.”

STB02: “I would never ask [Partner] how he was because I felt so guilty that three times my body had let us down and you know we hadn’t gone on to have that living child, so I didn’t wanna know how he was and then that caused a lot of issues between us.”

STB21: “This experience, the father is affected but the mother is the one who goes through the more complex aspects. First of all, because it is physical as well, so she has to do all the hard work before and after. I think the father is still an important figure when it comes to the support in these cases.”

STB09: “Then your relationships around you, it's not just your partner, but it's the whole family, it's such a ripple.”

**Outcomes related to older children**

STB18: “Oh [Baby’s name]’s going to miss Christmas’, and we sort of talked about how the Christmas before I’d been pregnant and then you know, [Baby’s name] wasn’t going to get to have Christmas with us, so she does... yeah she’s actually quite perceptive.”

STB08 “I think she was a bit confused, but I think at that age it’s quite matter of fact so we said the baby wasn’t big enough and strong enough to live outside of mummy’s tummy and unfortunately she died but you had a little sister and her name – we named her Meredith [0:23:01] and now she lives in heaven and that was pretty much as much as we needed to say. We kept it very matter of fact.”

STB21: “Our priority, for example, is our child. Of course [Baby’s name] is in our heart and we have tried to keep her alive.”

STB27: “No, and obviously we had [Son’s name] and [Daughter’s name] who were keeping us going all the time. RES1: We had to be up, we had to get them to school, they had their after-school activities. RES2: Yes, swimming, karate, piano lessons. RES1: All that was rolling on. RES2: Life drags you along.”

STB21: “Boss baby is a kid’s movie, and it is babies are to be with families, or babies come into this world to have a mission. When they end the mission, they need to go back to the baby’s world that is the sky. I used that movie, saying [Baby’s name] has been picked to be a manager! She was a boss, and she was born asleep. She had a mission here and her mission was to prepare my belly to have another, future baby, because she will not be able to be here in this world. He was sad, but was very calm, no crying, no shaking voice, very calm. He said, ‘so we need to wait another year?’ I said, ‘roughly’.

STB08: “She asked what would happen to her body if she’d gone, did her body go to heaven? I said, ‘Well no it didn’t.’ I wasn’t prepared to have the conversation about cremation with her so as far as she’s concerned, she’s buried because that felt less barbaric.”

STB10: “We have this thing that if we see a robin, we'll say thanks [Baby’s name] for sending the robin. So we saw one this morning when we were just at school and [Older daughter’s name] was like, oh there's a robin, thanks [Baby’s name] for sending the robin. So that's really nice. I love it when she talks about her.”

STB08: “I’ve shown her photos, so she’s seen her sister. She’s aware but she finds it very upsetting because it is, I get upset by it.”

STB04: “So, my daughter had a baby four years ago and then she didn’t want to have the baby at [Hospital name], even though it was nearer, she went to XXX as she couldn’t because the thought of you know… … the legacy of that, the impact of that goes on for long, long, long time, generations potentially.”

STB18: “I was like: ‘we have to tell her’. But she was kind of... acted a bit like me in that she was a bit like: ‘oh right, okay’ and she wasn’t like... when we told her about [Baby’s name] she was really excited, but she wasn’t excited this time. It was almost like she was kind of judging how she should feel from how we were behaving as well.”

STB27: ““If you need to go to the doctors or hospital will you come and pick us up from school, will you tell us? If there’s anything the matter will you tell us straight away?” So I think there’s definitely underlying issues from it.”

STB10: “I think because to begin with I found it quite difficult to concentrate on anything, including concentrating on playing with [Older daughter’s name], which I'd had no problem with before. So I've been much quicker to turn on the TV and stuff which I really feel guilty about.”

STB21: “The bereavement midwife said, ‘you are doing very well’. I said, ‘thank you, I believe so’. As I said, we have got a son. He needs to go to school every day. He needs to get his uniform clean. If it is not, they will send him home! He needs his packed lunch He needs to do his homework. He needs to go to swimming lessons. He has got more social life than us. He needs to continue going to all the birthday parties and all those things that he does. There shouldn’t be a bad impact on him.”

STB27: “Looking back at photos and we’re there and you can see smiles, but you know, you can just see that you’re not happy, you’re not there. Just going through the motions for the children really.”

STB08: “Everything, I worry when the wind blows that a power cable will come down and chop their heads off. I worry when they’re on the pavement that a car will mount the pavement and take us out or that a gust of wind will blow us into the path of a lorry or that the roof will collapse and squash them in their beds.”

STB27: “I’m saying there was no guidance as to how you should [involve children]. I think there was an immediate attention for [Mother’s name], very little on me and very little about how we then cope with our day to day lives, about kind of working forward with everybody.

**Future pregnancy and children outcomes**

**Outcomes related to planning a subsequent pregnancy**

STB14: “Yeah, I think straight after, even before the post-mortem, I overwhelmingly wanted to be pregnant again. It was strange because I didn’t want to replace her, but I’d been so prepared to be a mum and got used to the idea of having her here and, yeah, prepared our lives for her, bought her things, so there was quite an overwhelming drive.”

STB10: “I'm probably one of very few women that I've met who didn't actively want to get pregnant straightaway, because I don't really like being pregnant. I really... even though I really wanted a baby, I also very much wanted to grieve for [Baby’s name] and have time for [Baby’s name], because I really needed her to be part of our family and I didn't want another baby to be a replacement for her.”

STB06“I think having had a loss, like a stillbirth and then trying to conceive again, we felt very, very alone in that and it’s been emotionally so draining not getting further along and I think– there’s no one there that sits between this and the NHS.”

**Outcomes in a subsequent pregnancy**

STB12: “I think physically it felt quite hard been pregnant again, like it seemed like my pelvis were really painful and the consultant said well you’ve basically been pregnant for a year and a half and so your body’s not had any break… Yes and I suppose like stupid things are a bit annoying that like I definitely weigh a lot more than I did to start with and that must partly be because of having two pregnancies concurrent you know, I think I probably put a bit more weight on in my second pregnancy, so therefore I wouldn’t have had that if we’d had [Baby’s name] but like I definitely now know that worse has happened so and I definitely think we had got a much more different outlook on life now.”

STB25: “We were talking about dates and stuff, because we didn’t want to go – past 36 weeks was obviously always going to be horrific.”

STB18: “Yeah I’m planning to have an elective caesarean this time. I think partly because I got pregnant so quickly; I just feel I’m not... it was really traumatic. I don’t think I’m ready to go back to that, so yeah, so I’m going to have an elective caesarean and just so that it’s kind of calmer. I think what would be difficult for me going for a natural birth is, what if things start going wrong? Like if for example, the baby starts getting distressed or you know, ends up being induced and it doesn’t work, or if something like that happens, it would just be really stressful, so I just think, yeah, we’ve sort of opted to go for something planned where there’s a bit more control, for that reason.”

STB03: “The reassurance of hearing the heartbeat of the baby is very valuable, so that was really nice to … I’m always looking forward to that with the midwife and to give us the chance to hear the heartbeat of the baby. Initially it was very important when I couldn’t feel the baby kicking so that was very stressful initially but going and hearing the heartbeat that was very important.”

STB06: “I’d come away after the scan, I’d go back to work and I’d be absolutely shattered, it would be 10 o’clock in the morning and I’d drop you off at home, you’d go and have a nap, I was absolutely shattered. Mentally I’d worked myself up so much anticipating them saying, ‘I’m sorry, your baby is dead’ again. You’d come out and it was that relief, you’re happy, but the fatigue can get that way it just hits you, and for me that was the whole pregnancy.”

STB03: “I’m happy we’re pregnant again but I’m also obviously nervous because unlike previous other people we’ve met who’ve had miscarriages and various other things, they have a benchmark to get to, because once you get past the last point it went wrong, then you’re not … obviously you’re not completely safe, but you’ve gone beyond where it went wrong. Unfortunately, our benchmark is the day before the actual due date, so we’ve got a long time to go before we get there. So, I’ll be nervous until I’m holding a crying baby in my arms, and that’s the point I’ll know, ok, we’re not fully out of the woods yet, but we’ve gone beyond where things went wrong.”

STB02: “Yes we had one more, muffin. We’re adamant she was a little girl but again I’d literally just started working at my new job, you were in Liverpool weren’t you when I started bleeding and she just never developed but again I’d felt like the carrot had been dangled and then just ripped away and I struggled.”

STB09: “[Father’s name]’s opinion and he does help me with this to see another way and does it really matter because the end result, we’ve got a healthy baby boy. What more do we…? RES2: Yes. RES1: Whether he came out… RES2: However he comes out.”

STB10: “I guess the thing that I'm concentrating on most at the moment is my current pregnancy and this baby and being worried about whether this baby will survive or not. I guess the kind of pressure of being pregnant again.”

STB20;” Yeah, I felt horrendous but the minute I gave birth to [Baby’s name] I just wanted another baby. My ex-partner said all the time it absolutely was 100% not a replacement, we never wanted a replacement. I think when you spend nine months knowing that you are gonna have a baby at the end of it, it's like maternal instincts with all the hormones of giving birth as well, I just felt like I needed a baby, so I did know straight away and we tried as soon as periods came back, we were trying straight away again, because I think it only took a couple of months until they came back, so I did know straight away.”

STB13: “Yes. It’s ‘cause I never wanted any of the tests during the pregnancy. Well I don’t care; I just will love that child.”

STB12: “I think having the SANDS sticker on my notes was nice cause it meant that everyone kind of knows like, okay shits gone down, so kind of treat them nicely.”

STB08: “The midwife that had dealt with me and first told me that she couldn’t find a heartbeat bless her, came to my house to do my booking in appointment. I was only four weeks and three days. I’d only just found out I was pregnant, and she said I’m coming to you. You don’t need to come to the surgery, and she came and did my bloods and she bought me a plant and it’s beautiful.”

STB06: “Again, it was the same problem with the antenatal care that there was no continuity, so I felt like I was having to explain my story again and again and again. One of the things I’d read about on the Sands website, but I didn’t actually do anything about – I wish I had done – was that someone has invented stickers for notes that you can put on notes, so people know you’ve had that, but we didn’t have that, so I felt like every single time I was seeing someone different and being asked to explain my story.”

STB09: “I struggled a bit with the care that I was given from the midwives, just because of the continuity.”

STB17: “they used to put a sticker I think on the front of your folder. Sometimes when I used to go to my appointments, at the hospital mainly, because obviously as I say, the midwife here knew me, but it was always my scans and my hospital appointments with the consultant. They would open the folder and not even notice the sticker. Then I would have to, and they would say, “I’m really sorry.”

STB10: “I'd got stickers on the front of my notes. I've got one which is like a butterfly which is our Trust's way of saying I've had a previous pregnancy loss and I've also got one from Kicks Count, which actually says I've had a previous still birth on it. But when you're going in for scans and they quite often put a bit of paper over the front of the notes, so people don't see the stickers. So for my most recent scan I put a little note on the inside cover to say this is my third pregnancy, my second daughter [Baby’s name], died, please don't be afraid to use her name and talk about her.”

STB10: “Somebody who I had emailed to ask if she would look after me. So that's been very good from a continuity point of view. My midwife has been amazing. I just text her if I want to see her basically and she'll fit me in wherever. So those two people have been brilliant.”

“I've seen the obstetrician probably every three to four weeks and I've seen the midwife in-between times as well and she's scanned me each time she's seen... the obstetrician has scanned me each time she's seen me, which has been good.”

STB12: “So in my subsequent pregnancies he’d said as soon as you get pregnant again let me know and he saw us from about eight weeks and I had a section with my little girl who we had next and he came in and did the section and then at the end, he said uhm it won’t be me on the ward today ‘cause it’s actually my day off. So he’d come in to do our section so like he just is amazing in our mind.”

STB16: “Yes, we asked. It wasn’t her day, her day off and we’d still got contact with her and we said, “We’d really like you to be there. Would you like to be there?” I think the question, “We’d like you to be there,” probably answered. the question already and she was there, and it was nice to have her there. There was that connection, a friendly face if you like. As I said before, [Younger son’s name] was passed to me. He was put into a blanket and then put into a cot warmer that had got [Baby’s name]’s name on it, which was very nice, and it all went very well.”

STB19: “I didn't have to explain myself to her each time. I'd basically phone up and say, 'is [Doctor] there?' If [Doctor] wasn't there, I'd email her at work and say, 'I'm in the department can I pop up.' So she was phenomenal, she took a lot of my anxiety on board and understood it whereas if I'd had somebody different, they'd be like, 'oh so why are you having another scan? Why are you having this many scans for?' And I'd be like, 'well have you read my notes?' 'Oh yes, you've got one of those stickers on it.' So yes, [Doctor] was fantastic.”

STB21: “Is the same as I have done in my previous pregnancies, with [Son’s name], with [Baby’s name], with baby November and now, what do you want me to do? She is so excited. She is so happy. She called me yesterday and is more excited than somebody else I know. I am trying to keep calm. She is very happy, and I want to continue with her instead of picking another midwife.”

STB06: “But the scan, because when you’re going for the scan you can feel baby moving even when they’re doing the scan, immediately before having the scan, and there’s all that reassurance.”

STB11: “No, I had a scan with the consultant every two weeks, that was unusual. I was offered the blood flow scans as well, I can’t remember what they’re called, because of how [Baby’s name] died they realised that would be reassuring to see the blood flow from the cord to her. So they did quite a lot of detailed scans every time I went."

STB12: “I was part of a Tommy’s – the charity Tommy’s research clinic and they could do really specific specialised scans on my placenta and they found with my pregnancy, so our daughter called [Younger daughter’s name], so with my pregnancy at 24 weeks with [Younger daughter’s name] my placenta was the same size as it had been at 37 weeks with [Baby’s name] so that was really nice to know that that was growing, yes.”

STB17: “At that point for some reason I was obsessed with knowing that if I had another baby, I could have a caesarean, because I didn’t want to go through labour again, because my memory of labour was that and it was horrific. So he did me a letter to wherever I was having the baby that that would be okay.”

STB11: “Awful, the absolute worse pregnancy on earth. I wanted her to move constantly, and [Younger daughter’s name] was a very quiet baby which was a nightmare, because I did have to go to hospital a few times in a panic saying, “She’s not moving.” They scanned me every two weeks and they were very good.”

STB25: “I was there at the drop of a hat. I supposed I knew it annoys, when you turn up a lot, but it was so traumatising to do it. If they’d known how hard it was to physically get in there, and yet I would keep presenting. It was literally, I can’t tell you, I am so broken from that pregnancy, it was so hard, It was so hard, because I was just utterly convinced, like second to second, that she’s gonna die.”

STB06: “We were really anxious, and particularly because, as far as I knew, there was nothing wrong – there wasn’t any reason, there wasn’t anything we could avoid, and there weren’t really any kind of milestones.”

STB12: “I don’t know, I suppose I was anxious. I think I was anxious just when we were like on our way in for scans and things. I think I’d decided that I couldn’t live my life in a state of anxiety the whole time.”

STB18: “Yeah I felt like I was, you know I’d had three months of you know, being post-natal and grieving and planning the funeral and all of that, and I felt like I was just starting to get... feel a bit stronger, kind of physically – I joined the gym and I was exercising a bit and I’d started to feel a bit more, kind of like myself and then when I found out I was pregnant again it was like, it was almost like: ‘oh my god I’m back in this prison of being pregnant with kind of all the anxieties and, you know everything that goes along with being pregnant and not being able to do everything that you want to do, and feeling sick and all that’ and so it was... we were really lucky that it happened quickly but it was also really, really hard.”

STB18: “Not as extreme as that. I think in the first weeks afterwards – well certainly maybe the first month or so afterwards it was worst, but I have had various points in this pregnancy where I’ve, where I have felt really anxious, yeah, it’s a combination of anxiety and grief at the same time.:

STB12: “So I was like absolutely ecstatic and completely terrified probably in quite equal measure.”

STB20: “I just didn’t know what to do, I was just scared and although I wanted it I was scared and all these emotions and I just kept thinking well she was gonna die, I am not really gonna have a baby or I thought I was gonna have a miscarriage, I just thought something was gonna go wrong and I just knew I had to get out, I had to speak to people and it was more to just reassure myself I wasn’t crazy, that other people felt that way as well and it did reassure me because all the things that I was thinking that I thought were super abnormal, turned out to be the most common things that everyone else was thinking and feeling.

STB07: “So obviously since finding that we’re pregnant we have felt like we are moving forward now but it’s not easy ‘cause you’ve got the constant fear of something going wrong. It’s exhausting and I know that it’s gonna be a really difficult pregnancy ‘cause pregnancy is difficult anyway but after loss it’s you know a really harder process.”

STB18: “I mean I think, I think... you know I’ve always been really active and having two pregnancies more or less back-to-back has been really difficult. Obviously, I know I can stay active as well as pregnancy, but I’ve also had the kind of grief and general, you know, feeling low and depressed and unmotivated and probably not really in tune with my body.”

STB06: “Basically, the second pregnancy, when I had my first son who was born alive, the whole pregnancy was just pretty stressful I guess, because you just feel like something terrible is gonna happen at the end and you’re totally powerless to do anything about it.”

STB25: “All over it, like you know, ‘I’m a Rainbow Mum’ and I had a previous stillbirth all over my notes. INT: I’ll stick these on here. Do you think it helped with your mental health? RES2: Yeah, definitely. Yeah, 100%, and it was a laugh as well though.”

STB09: “After having [Subsequent baby], once I had finished breast feeding, it did start to rear its ugly head again. It wasn’t all plain sailing…I didn’t feel like he was my baby.”

STB19; “, I'd say for the first year we didn't really bond. I was a very, almost robotic Mum, you know, I was breastfeeding, also bottle feeding, she was very slow to gain weight, so it was almost like I was being pragmatic in, right I need to just try and get her weighed. We weren't really developing a relationship.”

STB01: “No the first one didn’t make me feel confident we’d ever have another baby at all. She said that there’s a 25% chance that this will happen again, that’s blatantly what she, she did say that there’s a 25% chance that this is going to happen again, and I was like oh.”

STB02: “Yes we’d just be promised more care and the percentage of it happening again was extremely low. I think she said about 10 percent, didn’t he?”

STB17: “I just thought I was in this hole that I was never going to get out of, and never going to have other children because I was struggling to conceive.”

STB25: “Then when I got pregnant again it just became like, you know, the meaning of life. Everything else came second. So, keeping the baby alive 24/7.”

STB20: “I just didn’t know what to do, I was just scared and although I wanted It, I was scared and all these emotions and I just kept thinking well she was gonna die, I am not really gonna have a baby or I thought I was gonna have a miscarriage, I just thought something was gonna go wrong.”

STB18: “I’ve had quite a lot of people, you know, say things like: ‘you know you’ve got a new baby coming now, so like that’s something to be really happy about’ or you know, which is true, but it also doesn’t take away from the grief that you’ve got about the baby that you’ve lost, and it can make you feel like your sadness is kind of inconvenient to them, or makes them feel uncomfortable.”

STB19: “So she was phenomenal, she took a lot of my anxiety on board and understood it whereas if I'd had somebody different, they'd be like, 'oh so why are you having another scan? Why are you having this many scans for?' And I'd be like, 'well have you read my notes?' 'Oh yes, you've got one of those stickers on it.'”

STB03: “I’m still following my counsellor now. So, I’m currently 24 weeks pregnant, so it’s a lot of anxiety, underlying the pregnancy and it’s good to have maybe not as often counselling session as I used to at the beginning, but having a catch-up once a month is very useful for me, so, to go through the process of and managing the anxiety, it’s very useful.”

STB19: “INT: And what other benefits do you think you've had from the Sands group?

RES: It helped me carry [Baby number 2’s name], I think all my anxiety through carrying her helped me a lot.”
