# Supplementary material for: What outcomes are important to families with a lived experience of stillbirth? A qualitative study to inform the development of a core outcome set for stillbirth care
Source: PLoS One. 2026 May 19;21(5):e0347544. doi: 10.1371/journal.pone.0347544 (PMC13186333; doi:10.1371/journal.pone.0347544)

Figure 1: Flow chart of data collection, coding and analysis process for qualitative interviews

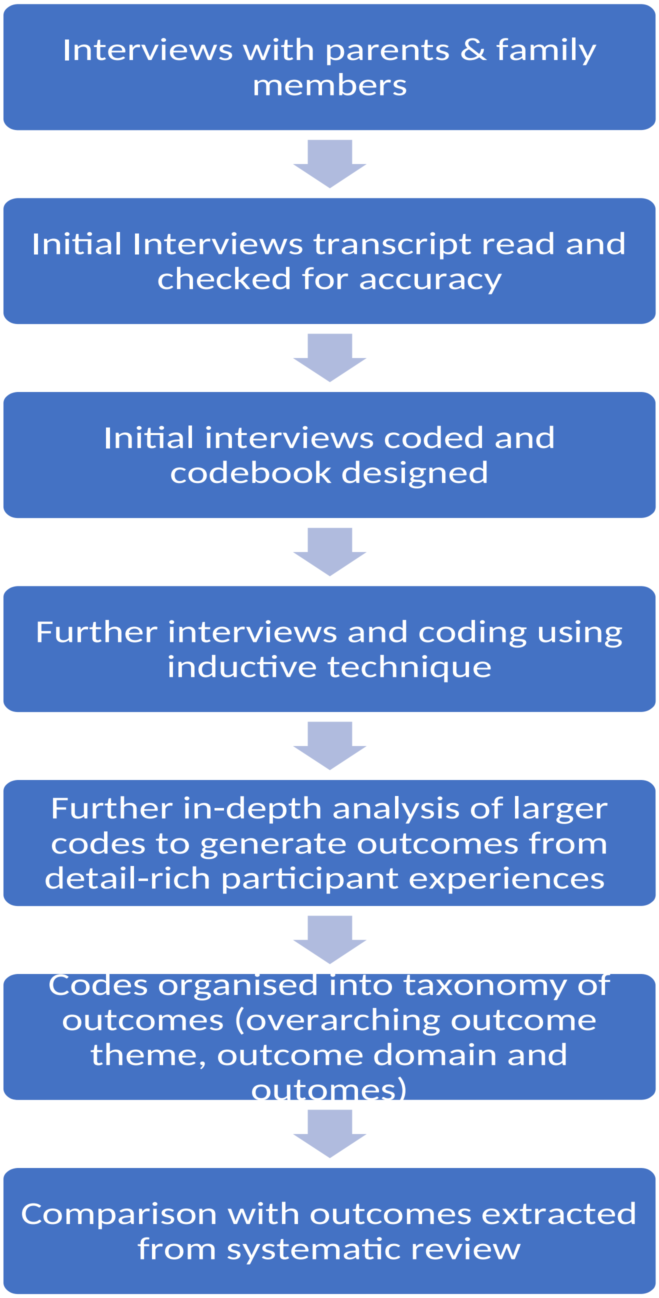

Supplement: Supplementary file 6 [file pone.0347544.s003.docx]
