# Supplementary material for: What outcomes are important to families with a lived experience of stillbirth? A qualitative study to inform the development of a core outcome set for stillbirth care
Source: PLoS One. 2026 May 19;21(5):e0347544. doi: 10.1371/journal.pone.0347544 (PMC13186333; doi:10.1371/journal.pone.0347544)
Supplement: Attachment — Submitted filename: Revision_manuscript PONE-D-25-09270.docx [file pone.0347544.s007.docx]

**Revision of the manuscript PONE-D-25-09270**

**What outcomes are important to families with a lived experience of stillbirth? A qualitative study to inform the development of a core outcome set for stillbirth care**

I would like to begin by congratulating the authors on addressing such an important and sensitive topic. The focus on identifying outcomes that are important to families with lived experience of stillbirth is highly relevant both to the advancement of research in this area and to improving care practices for families affected by such a devastating event. The study’s aim—to inform the development of a core outcome set for stillbirth care based on the perspectives of families—is both timely and essential, ensuring that research and interventions in this field are aligned with the needs and priorities of those most directly impacted.

That said, despite the relevance and potential contribution of the manuscript, there are several major issues that require careful revision before the manuscript can be considered for publication. These issues pertain particularly to the framing of the study within existing literature, the clarity of methodological procedures, and the depth of analysis and discussion of the results.

**Contextualization and Literature Review**

The authors argue that their qualitative study identified potential care outcomes, the majority of which had not been previously reported in stillbirth care studies. This is a crucial contribution. However, the manuscript would benefit from a more thorough integration of prior literature on outcomes related to stillbirth care. While the authors refer readers to complementary sources for further context, given the centrality of this point to the value of the study, the introduction should provide a more self-contained and explanatory overview of what is already known in the field. Doing so would better contextualize the originality and significance of the study’s findings, and more clearly highlight the added value of the present work.

**Methodological Clarity**

Further clarification is needed regarding the sampling and recruitment processes. For instance:

- How were different family members contacted?
- Who facilitated these contacts?
- Were all interviews conducted individually?
- Despite outreach to various family members, only 26 mothers and 12 fathers participated. Were other relatives invited? If so, how many, and what reasons were given for declining to participate?
- Beyond the eligibility criteria described, were there any additional exclusion criteria?

The description of data collection and analysis also requires additional detail. It is unclear how theoretical saturation was determined—was it based on the outcomes themselves or on the codes within these outcomes? Similarly, when the authors state that 298 out of 343 potential outcomes had not been previously reported, is this count based on final outcomes or on individual codes?

**Results Presentation**

In the results section, while the outcome domains are described, it would greatly strengthen the manuscript to indicate how many participants mentioned each outcome. This would help readers gauge the relative importance or salience of each domain.

Specific clarifications are also needed in several areas:

- Under **Care Experience**, the authors write: “A number of parents were concerned about the emotional impact that providing stillbirth care had on staff and thus this was identified as an outcome.” This statement is unclear. What exactly did participants mean by this, and how does it translate into an outcome of care?
- Regarding outcomes in the context of **multiple pregnancies**, how many participants experienced a stillbirth in this specific context?
- In the **Grief Outcomes** section, the authors note differences in how men and women grieve. What were these differences, and how were they manifested?
- Within **Mental Health Outcomes**, the text refers to various types of interventions. Were these interventions actually received by participants, or were they interventions the participants wished they had received?
- The same question applies to the **Social Outcomes** section—are these based on experienced support or unmet needs?
- Concerning **Emotional Health Outcomes**, where participants mentioned positive emotions such as optimism and pride, it would be important to clarify what these emotions were associated with (e.g., personal growth, support received, subsequent parenting experiences).
- The **Partner and Family Relationship Outcomes** domain would benefit from further detail regarding how relationships were affected. The current description is too vague.
- For **Outcomes Related to Older Children**, was the impact described as positive, negative, or mixed? More concrete detail is needed.
- The **Outcomes Relevant to Subsequent Children** domain also requires further elaboration in order to understand the full scope and significance of the findings.

**Discussion and Interpretation**

The discussion section appears underdeveloped and overly focused on narrow or specific points. A more comprehensive discussion of the broad range of outcome domains that emerged from the interviews is warranted. In particular, I would have appreciated a deeper comparative analysis between the findings of this qualitative study and those of the authors’ previously conducted systematic review. The discrepancy between these two sources should be examined further: how do the authors explain this divergence? Such an analysis could shed light on potential gaps between research, practice, and the actual needs and perspectives of affected individuals.

In the **Strengths and Limitations** section, the authors note that a particular effort was made to ensure diversity of experiences, with participants having experienced stillbirths as recently as one year ago and as far back as 50 years. While this breadth is indeed a strength, it would be valuable to explore whether the timing of the experience influenced the outcomes reported. Similarly, did mothers and fathers report different outcomes? Were any other sociodemographic or contextual differences observed in the nature or frequency of outcomes?

Addressing these points would allow the authors to offer more tailored insights for designing research and interventions that are responsive to the varied needs of specific subgroups within this population.

**Conclusion**

In sum, this manuscript represents an important and timely contribution to stillbirth research, with the potential to meaningfully inform both policy and clinical practice. However, several major issues must be addressed—particularly in terms of contextual framing, methodological transparency, and the breadth and depth of discussion—before this manuscript can be considered ready for publication.

Thank you for the opportunity to review this important work.
