# Supplementary material for: What outcomes are important to families with a lived experience of stillbirth? A qualitative study to inform the development of a core outcome set for stillbirth care
Source: PLoS One. 2026 May 19;21(5):e0347544. doi: 10.1371/journal.pone.0347544 (PMC13186333; doi:10.1371/journal.pone.0347544)
Supplement: Attachment — Submitted filename: Response to reviewers.docx [file pone.0347544.s008.docx]

Dear Plos One,

Thank you for considering our manuscript following revisions. Please see response below:

| **Reviewer comment** | **Response to reviewer** |
| --- | --- |
| Please ensure that your manuscript meets PLOS ONE's style requirements, including those for file naming. | We have edited manuscript according to PLOS ONEs style requirements. |
| Please provide an amended statement that declares *all* the funding or sources of support (whether external or internal to your organization) received during this study, as detailed online in our guide for authors at http://journals.plos.org/plosone/s/submit-now.  Please also include the statement “There was no additional external funding received for this study.” in your updated Funding Statement.  Please include your amended Funding Statement within your cover letter. We will change the online submission form on your behalf. | The following statement has been amended.  This report is an independent research arising from a doctoral fellowship (DRF) supported by the National Institute for Health Research (NIHR DRF-2017-10-130). The views expressed in this publication are those of the authors and not necessarily those of the National Health Service, the National Institute for Health Research or the Department of Health. There was no additional external funding received for this study. |
| In the online submission form, you indicated that [All relevant data are within the manuscript and its Supporting Information files. Full data cannot be shared publicly due to confidentiality and GDPR. Data are available from the University of Oxford for researchers who meet the criteria for access to confidential data.].  All PLOS journals now require all data underlying the findings described in their manuscript to be freely available to other researchers, either 1. In a public repository, 2. Within the manuscript itself, or 3. Uploaded as supplementary information.  This policy applies to all data except where public deposition would breach compliance with the protocol approved by your research ethics board. If your data cannot be made publicly available for ethical or legal reasons (e.g., public availability would compromise patient privacy), please explain your reasons on resubmission and your exemption request will be escalated for approval. | Full access to interview transcripts would breach compliance with the protocol approved from the ethics board and cannot be made publicly available as it would compromise anonymity and patient privacy. |
| We note that you have provided funding information that is currently declared in your Funding Statement. However, funding information should not appear in the Acknowledgments section or other areas of your manuscript. We will only publish funding information present in the Funding Statement section of the online submission form.  Please remove any funding-related text from the manuscript and let us know how you would like to update your Funding Statement. Currently, your Funding Statement reads as follows: | The funding statements have been removed from the manuscript. |
| Please confirm that this does not alter your adherence to all PLOS ONE policies on sharing data and materials, by including the following statement: "This does not alter our adherence to  PLOS ONE policies on sharing data and materials.” (as detailed online in our guide for authors <http://journals.plos.org/plosone/s/competing-interests>).  If there are restrictions on sharing of data and/or materials, please state these. Please note that we cannot proceed with consideration of your article until this information has been declared.  Please include your updated Competing Interests statement in your cover letter; we will change the online submission form on your behalf. | The following statement has been added to disclosures of interest.  DB is Trainee Scientific Editor for BJOG. AEPH is the Director of the Tommy’s Stillbirth Research Centre, University of Manchester which receives grant funding for stillbirth research. This does not alter our adherence to  PLOS ONE policies on sharing data and materials. |
| Please amend the manuscript submission data (via Edit Submission) to include author Lisa Hinton | This has been amended to include Lisa Hinton. |
| One of the noted authors is a group or consortium [iCHOOSE Collaborative Group]. In addition to naming the author group, please list the individual authors and affiliations within this group in the acknowledgments section of your manuscript. Please also indicate clearly a lead author for this group along with a contact email address. | The group has been listed in the acknowledgements and lead author and email included. |
| Your ethics statement should only appear in the Methods section of your manuscript. If your ethics statement is written in any section besides the Methods, please move it to the Methods section and delete it from any other section. Please ensure that your ethics statement is included in your manuscript, as the ethics statement entered into the online submission form will not be published alongside your manuscript. | The ethics statement has been moved to the methods section. |
| Please include a separate caption for each figure in your manuscript. | Separate captions have been included for each figure. |
| Please include captions for your Supporting Information files at the end of your manuscript, and update any in-text citations to match accordingly. Please see our Supporting Information guidelines for more information: [http://journals.plos.org/plosone/s/supporting-information](http://track.editorialmanager.com/CL0/http:%2F%2Fjournals.plos.org%2Fplosone%2Fs%2Fsupporting-information/1/010f01977d05adba-bda47d9a-0bd2-4e4d-8ed0-5ff8528e0df7-000000/W82VgWwE121VL0DmvlfKGPj2plCTB3d74LJDh2uvTJE=215). | Captions for supporting information have been included at the end of the manuscript. |
| Please include your tables as part of your main manuscript and remove the individual files. | Tables have been now included in the main manuscript. |
| I would like to begin by congratulating the authors on addressing such an important and sensitive topic. The focus on identifying outcomes that are important to families with lived experience of stillbirth is highly relevant both to the advancement of research in this area and to improving care practices for families affected by such a devastating event. The study’s aim—to inform the development of a core outcome set for stillbirth care based on the perspectives of families—is both timely and essential, ensuring that research and interventions in this field are aligned with the needs and priorities of those most directly impacted. | Thank you for this compliment. |
| That said, despite the relevance and potential contribution of the manuscript, there are several major issues that require careful revision before the manuscript can be considered for publication. These issues pertain particularly to the framing of the study within existing literature, the clarity of methodological procedures, and the depth of analysis and discussion of the results. | We have addressed your feedback by revising the manuscript as stated below. We hope this improves the framing of the study within the existing literature, clarity and depth of the results. |
| The authors argue that their qualitative study identified potential care outcomes, the majority of which had not been previously reported in stillbirth care studies. This is a crucial contribution. However, the manuscript would benefit from a more thorough integration of prior literature on outcomes related to stillbirth care. While the authors refer readers to complementary sources for further context, given the centrality of this point to the value of the study, the introduction should provide a more self-contained and explanatory overview of what is already known in the field. Doing so would better contextualize the originality and significance of the study’s findings, and more clearly highlight the added value of the present work. | Thank you for this feedback, we have now inserted more detail about the types of outcomes reported in previous studies to give more context. Please see page 5 lines 9 to 15.  “Outcomes reported in previous studies of stillbirth care, have primarily focused on medical outcomes, negating the wider impacts of stillbirth on parents^4^. For example, findings from the same systematic review found no studies on labour and birth care reporting psychological outcomes such as grief or anxiety. Similarly, no studies evaluating bereavement hospital care considered outcomes relating to parents’ role, for example, returning to work or caring for older children^”^ |
| Further clarification is needed regarding the sampling and recruitment processes. For instance:   1. How were different family members contacted? 2. Who facilitated these contacts? 3. Were all interviews conducted individually? 4. Despite outreach to various family members, only 26 mothers and 12 fathers participated. Were other relatives invited? If so, how many, and what reasons were given for declining to participate? 5. Beyond the eligibility criteria described, were there any additional exclusion criteria? | More information about the sampling and the recruitment is included   1. Interested family members emailed the research team to arrange a potential interview (Page 8 line 9-10). 2. DB (project lead) facilitated recruitment into the study (Page 8 line 10). 3. Parents were interviewed individually or jointly (with partner or family member). (Page 9 line 2). 4. Yes, other relatives were invited including bereaved siblings and grandparents, due to the nature of recruitment approach we are unable to ascertain the total number of family members invited to participate and therefore a response rate or reasons for declining is not documented. (Page 8 line 7-11). Two bereaved siblings participated in the interviews. (Page 11 line 3). 5. No further exclusion criteria were applied. |
| The description of data collection and analysis also requires additional detail. It is unclear how theoretical saturation was determined—was it based on the outcomes themselves or on the codes within these outcomes? Similarly, when the authors state that 298 out of 343 potential outcomes had not been previously reported, is this count based on final outcomes or on individual codes? | Thank you for this comment. Theoretical saturation was determined when no new outcomes or themes emerged (not codes). More detail is provided in the manuscript Page 8 & 9 lines 20-21 & 1&2  “Recruitment and interviews continued until it was considered that theoretical saturation was approaching (i.e. no new themes or outcomes emerged). To ascertain if the sample size was sufficient, dimensions of information power were also considered including; study aim, sample specificity, use of established theory, quality of dialogue and analysis strategy.”  The final list was based on final outcomes not individual codes. See supporting information 5 and figure 1. Also, page 10 lines 15-18.  “Codes were grouped (where relevant) and experiences translated into outcomes through discussion with the research team. Similar outcomes were arranged into outcome domains, which were then organised into four overarching care outcome themes. **See Figure 1 and S3: Figure 1.** Outcomes generated were cross-referenced against the outcomes extracted from our systematic review on interventions and outcomes in stillbirth care research. “ |
| In the results section, while the outcome domains are described, it would greatly strengthen the manuscript to indicate how many participants mentioned each outcome. This would help readers gauge the relative importance or salience of each domain. | Thank you. Figure 2 now contains number of interviews mentioning each outcome domain. |
| Under **Care Experience**, the authors write: “A number of parents were concerned about the emotional impact that providing stillbirth care had on staff and thus this was identified as an outcome.” This statement is unclear. What exactly did participants mean by this, and how does it translate into an outcome of care? | Thank you, this statement has now been revised and expanded for more context. Page 13 line 4-6.  “A number of parents were concerned about the impact of providing stillbirth care on healthcare professionals; one parent described a midwife handing in her notice another recalled a student midwife “crying floods of tears” after her baby was born.” |
| Regarding outcomes in the context of **multiple pregnancies**, how many participants experienced a stillbirth in this specific context? | Three sets of parents who had experienced a stillbirth in a multiple pregnancy and a surviving twin sibling were interviewed. This is now included on Page 13 line 16-17. |
| In the **Grief Outcomes** section, the authors note differences in how men and women grieve. What were these differences, and how were they manifested? | Thank you, have now expanded the section on grief outcomes to include more about the differences in how men and women grieve. Page 14 Line 4-13.  “Common feelings related to overwhelming grief were shock, despair, emptiness, pain, feeling lost, hopelessness, anger and denial. As part of their grief many parents, described feeling guilty and a sense of failure. Mothers described blaming themselves for the death of their baby or wondering if they could have prevented the and so the investigations to understand why a baby died were instrumental in helping to resolve these feelings. Fathers described keeping their feelings to themselves or dealing with their grief at a later point compared with their partners, as they focused on practical tasks such as arranging the funeral and supporting their family. One father did not “grieve properly” in the first year which led to panic attacks and physical symptoms. Fathers often were not offered support by healthcare professions or did not seek support until many years later to help process their grief.” |
| Within **Mental Health Outcomes**, the text refers to various types of interventions. Were these interventions actually received by participants, or were they interventions the participants wished they had received? | These were interventions received by participants. The following sentences have been revised to provide more clarity. Page 14 Lines 17-20.  “Types of mental health intervention received included cognitive behavioural therapy, mindfulness, pharmacological treatment and eye movement desensitisation and reprocessing (EMDR) for PTSD. Experiences of counselling was discussed by most and reported to have many mental health benefits...” |
| The same question applies to the **Social Outcomes** section—are these based on experienced support or unmet needs? | These were types of coping support used by parents to help their feelings of isolation. The following sentence has been revised on Page 16 line 1.  “Several coping interventions were utilised by parents, to help alleviate the feeling of isolation...” |
| Concerning **Emotional Health Outcomes**, where participants mentioned positive emotions such as optimism and pride, it would be important to clarify what these emotions were associated with (e.g., personal growth, support received, subsequent parenting experiences). | We have clarified what positive emotions were associated with on Page 15 line 8-10.  “Conversely positive emotions were also described, such the sense of pride they felt giving birth to their baby and seeing them for the first time. One parent recalled feeling more optimistic after psychological therapy and another had more hope in a subsequent pregnancy when they had specialist care from their hospital.” |
| The **Partner and Family Relationship Outcomes** domain would benefit from further detail regarding how relationships were affected. The current description is too vague. | This domain is further expanded on Page 16 Lines 11-25 and Page 17 Line 1-2 to detail further on how relationships were affected.  “Parents reported varying degrees in which their relationships with their partner were affected following the stillbirth. Several described the death of their baby strengthening their relationship and bringing them closer together as a couple. One mother felt that she and her husband dealt with things differently to begin with, however talking about their baby girl helped their relationship, this was facilitated by relationship counselling for some parents. For others it had a negative impact leading to relationship difficulties, arguments, separation or divorce. Trying to become pregnant again soon after the stillbirth led to further challenges and “pressure” to some relationships, particularly when it took longer than expected to conceive.  The impact on relationships in the family were important. According to those interviewed, family members such as grandparents and siblings grieved for the baby and found the emotional impact difficult. Having support and a family network helped many parents with their grief. Friends were also important as a source of support in challenging times and many appreciated people wanting to spend time with them and talk about their stillborn baby. Although friends and family wanted to offer support, sometimes they did not know what to say or do. This left some parents feeling very alone. Some parents described losing friendships when friends were not there for them. Others gained friendships after the stillbirth or found comfort in friends who had also experienced the loss of a baby in pregnancy.” |
| For **Outcomes Related to Older Children**, was the impact described as positive, negative, or mixed? More concrete detail is needed. | More detail is included on the impact on older children on Page 17 Line 5-22.  “All parents felt it was important that their children were aware of what happened. However, they differed on their preferences on whether to introduce their baby to their children. Some parents decided to not allow their children to meet the baby, either due to the overwhelming shock or the perception that the children were too young at the time to cope. Whilst other older children, decided to meet the baby out of their own choice. Including the children in the memory making process, allowing them to hold, spend time and take photographs was a positive experience for one family, however their six-year-old was upset when they had to say goodbye to their sibling.  Some parents reported no significant effects of the bereavement on their children, whilst others described more long-lasting negative impacts on their mental health and behaviour, including separation anxiety and stress. In the short term, some parents described finding it difficult to concentrate on their children. Whilst for others, older children provided a distraction, allowing some parents to keep going despite their grief. Parents wanted to try and keep everything as normal as possible for their children, by keeping their daily routines. Parents described being more anxious, cautious or over-protective following the stillbirth. Parents described seeking support for children in a variety of ways, including from healthcare professionals such as the bereavement midwife, child bereavement charities, counselling, school or support books written to help parents discuss the stillbirth with their children.” |
| The **Outcomes Relevant to Subsequent Children** domain also requires further elaboration in order to understand the full scope and significance of the findings. | Further elaboration is included in the outcomes relevant to subsequent children domain. Page 19 Line 8 to 13.  “Some mothers described a delayed grief reaction or postpartum depression which made it hard for them to bond with their new baby in a subsequent pregnancy. A few mothers recalled feeling very frightened and worried about whether their baby would survive the newborn period because of experiencing a stillbirth in the past. This led to more attendances to the doctors due to anxiety over their baby’s health. Having empathic support with parenting from care professionals such as the health visitor was seen as crucial to help them cope.” |
| The discussion section appears underdeveloped and overly focused on narrow or specific points. A more comprehensive discussion of the broad range of outcome domains that emerged from the interviews is warranted. In particular, I would have appreciated a deeper comparative analysis between the findings of this qualitative study and those of the authors’ previously conducted systematic review. The discrepancy between these two sources should be examined further: how do the authors explain this divergence? Such an analysis could shed light on potential gaps between research, practice, and the actual needs and perspectives of affected individuals. | Thank you further in-depth discussion is including on the broad range of outcomes including, grief (Page 22 Lines 3-10, outcomes related to the whole family (Page 23 Lins 7-16), future family (Page 23 Lines 18-24), subsequent pregnancy (Page 23 Lines 26-28 & Page 24 1-7). Divergences and gaps in research are discussed.  “All parents interviewed experienced grief in one form or another, yet their lived experience of this grief was individual. Grief alone is not a unique outcome several studies have measured grief when evaluating care after stillbirth^4,34–39^. What was most interesting was the multitude of facets of grief that could be measured as identified from the analysis of the interviews, for example overwhelming grief, coping with grief, feelings self-blame, guilt and failure. Any number of these could be measured in a future study, and as such we need to understand what is most important to measure from the perspectives of parents, healthcare professionals and researchers, so as we are able to use the correct measurement tools.  For parents, outcomes that related to the whole family were imperative. Measuring the outcomes related to the impact of the whole family, such as partner, siblings, older children had special significance to parents. The systematic review found that no quantitative studies measuring the impact on children or parenting, yet many parents spoke of the significant psychological impact that the stillbirth had on their children^4^. Several families introduced their children to their baby and involved them in the memory making process. There is mixed evidence on the psychological impact of seeing, holding and spending time with the baby in parents, with conflicting evidence on whether there is a positive or negative effect on PTSD or depression^4^. Further research is required to ascertain the psychological consequences of involving older children in the memory making process, and consideration will have to be made on the feasibility of measuring longer-term outcomes involving family members.  Outcomes connected to the future family were also important to parents. The interviews identified some novel outcomes relevant to planning a subsequent pregnancy including perceived support for planning a subsequent pregnancy, reassurances about fertility and requiring fertility treatment and support. The previous systematic review identified no research studies on interventions or outcomes related specifically to planning a subsequent pregnancy or pre-pregnancy counselling^4^. Findings from these interviews appear to suggest that additional pre-pregnancy support would be appreciated by parents considering planning a subsequent pregnancy following their stillbirth.  Seventy-nine new outcomes were identified specifically related to a subsequent pregnancy, that have not been measured by researchers in the systematic review. The overall concern of the parents who went on to have subsequent pregnancies was of the survival of the baby, and therefore a core outcome should acknowledge this appropriately. A holistic range of outcomes were identified from the interviews including medical, social, psychological and experiential outcomes, whilst previous research into care in a subsequent pregnancy has focused on medical outcomes as opposed to the full range of possible outcomes. When comparing with the systematic review, additional outcomes were also identified from the interviews relevant to maternal and fetal health, hospital resource use outcomes (e.g., additional hospital appointments, additional scan appointments) , bereavement support and care that recognises previous stillbirth^4^.” |
| In the **Strengths and Limitations** section, the authors note that a particular effort was made to ensure diversity of experiences, with participants having experienced stillbirths as recently as one year ago and as far back as 50 years. While this breadth is indeed a strength, it would be valuable to explore whether the timing of the experience influenced the outcomes reported. Similarly, did mothers and fathers report different outcomes? Were any other sociodemographic or contextual differences observed in the nature or frequency of outcomes?  Addressing these points would allow the authors to offer more tailored insights for designing research and interventions that are responsive to the varied needs of specific subgroups within this population. | Further discussion on the types of outcomes reported in relation to time and mothers and fathers are discussed on Page 20&21 (Line 17-2).  “Interviews were conducted with mothers and fathers, as it was important to consider both parents’ experiences. Similar outcomes were reported; however, fathers often spoke of the focus being on the mother in the early days and the lack of professional and social support after the death of their baby. This led to negative mental health outcomes and physical symptoms of grief for some fathers. Future research should focus on bereavement care interventions that include fathers as participants to improve psychological wellbeing.”  “A possibly limitation is recall and the differences in care experienced by parents at that time, specifically the lack of bereavement care, counselling and support. Despite this many similar outcomes emerged from the analysis for example grief, stigma and isolation. By taking a longer-term perspective, the interviews were able to capture outcomes related to returning to work and future pregnancies.” |
| In sum, this manuscript represents an important and timely contribution to stillbirth research, with the potential to meaningfully inform both policy and clinical practice. However, several major issues must be addressed—particularly in terms of contextual framing, methodological transparency, and the breadth and depth of discussion—before this manuscript can be considered ready for publication.  Thank you for the opportunity to review this important work. | Thank you, we hope the changes above address these issues satisfactorily. |
| Reviewer 2  I congratulate the authors and their interest in these topics, which are so necessary and pertinent. Parents and families who experience stillbirth need special attention and professional care. The article is well organized and adequate in the way the results are presented. The themes and categories are well represented and are in line with recently published studies. However, some improvements could be made to make the study clearer and more objective. Namely, in introduction, method and results. | Thank you we have improved the introduction, methods and results for more clarity and in response to comments above, |
