# Supplementary material for: What outcomes are important to families with a lived experience of stillbirth? A qualitative study to inform the development of a core outcome set for stillbirth care
Source: PLoS One. 2026 May 19;21(5):e0347544. doi: 10.1371/journal.pone.0347544 (PMC13186333; doi:10.1371/journal.pone.0347544)
Supplement: Attachment — Submitted filename: Response_to_Reviewers_auresp_2.docx [file pone.0347544.s009.docx]

Centre for Academic Women’s Health

School of Translational Health Sciences

University of Bristol

Southmead Hospital

Westbury on Trym
Bristol

BS10 5NB

United Kingdom

10^th^ March 2026

Dear PLOS One,

Please find enclosed our second revised manuscript entitled “**What outcomes are important to measure for stillbirth care research? A qualitative study of the lived experience of stillbirth to inform the development of a core outcome set”** by Bakhbakhi et al. We hope our amendments make this manuscript suitable for publication in PLOS One. Please see response to reviewers for all edits to manuscript and responses to reviewers.

**Please see response to reviewer’s comments below:**

1. On page 9, line 6, there appears to be a typo: I believe a comma (,) rather than a semicolon (;) should follow “gestation or more;”.

**This has been amended accordingly in the manuscript.**

2. On page 10, the authors note that interviews were conducted either individually or together with the partner or another family member. I would kindly suggest including some brief considerations regarding whether this may have influenced participants’ behavior during the interviews, how coding was managed when information came from different participants within the same interview, and whether such circumstances had any impact on the results.

**Thank you for this helpful suggestion.**

**We have added this as a potential limitation in the discussion on Page 26 Line 14-15. “Furthermore, some interviews were conducted together with the partner, this may or may not have influenced what a parent chose to share or emphasise, and we note this as potential limitation.”**

**We have also clarified the coding approach in the methods as follows on Page 11 Line 21-23**

**“Each participant’s contributions/outcomes, whether from the bereaved mother, partner or family member were coded individually and each quotation presented in the results is attributed to the specific parent who said it. ”**

**In addition, we have updated Table 2. Outcome themes and domains with illustrative participant quotes to specify which parent or family member contributed to each quote, providing greater transparency.**

3. In the Results section, when presenting the outcome themes and domains, there are still some instances of vague expressions such as “several parents,” “several mothers,” “a number of parents,” “many parents,” or “by most.” I encourage the authors to replace these with objective quantitative indicators, specifying how many participants actually made those references.

**Thank you for highlighting this. We have carefully revised the Results section to replace vague descriptors (e.g., “several,” “many,” “a number of parents”) with specific counts indicating how many participants raised each outcome. This provides greater clarity and transparency regarding the distribution of views.**

**At the same time, we have avoided providing exact numerical figures in every instance to remain aligned with established qualitative research principles, which emphasise depth of understanding rather than quantification. As noted by Green and Thorogood in Qualitative Methods for Health Research (Sage, 2018), qualitative enquiry aims to explore “what, how, or why” rather than measure phenomena, and the data generated are typically in the form of words rather than numbers. We therefore report precise participant numbers where appropriate while maintaining fidelity to qualitative methodology.**

4. In the Discussion section, on page 26, I recommend that the authors not only emphasize the importance of including both parents but also discuss the relevance of allowing participation of other family members, highlighting the value this broader perspective may bring.

**Thank you for this suggestion. We have included this statement in the discussion “An important strength of this study was that an inclusive approach was adopted, including both parents and participation of other family members in the interviews, thus broadening the scope of outcomes identified.”**

5. I suggest introducing the final paragraph before the Conclusion with the phrase “Finally,” to strengthen the flow of the argument.

**Thank you this has been added to page 30 line 13.**

6. Lastly, the article would benefit from a more comprehensive Conclusion section, providing greater detail and reinforcing the contributions and value of this work.

Thank you a more comprehensive conclusion section has been included. See Page 30 line 13-21.

**“Finally, this in-depth qualitative study of the lived experience of stillbirth has identified a wide range of novel outcomes, providing a comprehensive and nuanced understanding of the outcomes that matter most to families. By translating the lived experience of stillbirth into potential outcomes, a broad range of impacts were identified, many of which have been overlooked and under-measured in previous stillbirth care research. Importantly, the outcomes span clinical, psychological, social and future pregnancy domains demonstrating the need for a holistic approach to evaluating care. These outcomes have enriched the long-list of outcomes in the next stage of developing a core outcome set for stillbirth care research, ensuring that the priorities and voices of bereaved parents are embedded in final core outcome set. A core outcome set grounded in parental and family insights will help improve the relevance and impact of future research for more effective and compassionate care after stillbirth.”**

7. In summary, this is a much-improved manuscript that makes a valuable contribution to the field. For the reasons outlined above, I am favorable to its publication in its current form, once the minor issues highlighted are addressed.

**Thank you.**
